# Fragment-Specific Stable Carbon Isotope Analysis for Screening the Authenticity of Red Yeast Rice Products

**DOI:** 10.3390/foods15203677

**Published:** 2026-10-16

**Authors:** Zhuying Wu, Daobing Wang, Di Feng, Yuan Shuang, Shanshan Zhao, Xueyao Li, Yuhan Tang, Guohui Li, Lanqing Wang, Qiding Zhong, Cuiying Zhang

**Affiliations:** 1 College of Biotechnology, Tianjin University of Science and Technology, Tianjin 300457, China; zhuyingwu365@126.com; 2 Technology Innovation Center of Light Industrial Consumer Goods Quality and Safety for State Administration for Market Regulation, Sinolight Technology Innovation Center Co., Ltd., Beijing 100015, China; 3 College of Chemistry and Life Science, Beijing University of Technology, Beijing 100022, China; 4 Sinolight Inspection and Certification (Taiyuan) Co., Ltd., Taiyuan 030400, China

**Keywords:** red yeast rice, monacolin K, lovastatin, 2-methylbutyric acid, carbon isotope ratio, LC-IRMS, food authentication

## Abstract

Monacolin K (MK) in red yeast rice (RYR) is structurally identical to pharmaceutical lovastatin in its lactone form, making their origins difficult to distinguish. This study examined the δ^13^C value of hydrolysis-derived 2-methylbutyric acid (2-MBA) as a fragment-specific screening marker using liquid chromatography–isotope ratio mass spectrometry (LC-IRMS). Within-day repeatability was 0.19‰ for RYR, interlaboratory standard deviations were 0.18–0.19‰, and a two-component expanded uncertainty estimate was ±0.58‰ (*k* = 2). Hydrolysis-derived 2-MBA ranged from −32.73‰ to −26.68‰ in 45 authentic RYR samples and from −10.17‰ to −6.87‰ in 23 pharmaceutical lovastatin batches. Controlled blends showed a linear relationship between δ^13^C and blend ratio (R^2^ = 0.9997). A one-sided tolerance limit at 95% confidence and 99% population coverage gave an investigational screening threshold of −26.47‰ for the sampled Chinese authentic RYR population. Three of 20 commercial products exceeded this threshold and were classified as isotopically inconsistent with that population. Overall, this study developed and analytically evaluated a fragment-specific LC-IRMS approach for screening source-related isotope differences between RYR-derived MK and pharmaceutical lovastatin under the investigated conditions, providing a potential analytical tool for RYR authenticity assessment.

## 1. Introduction

**Figure 1:**
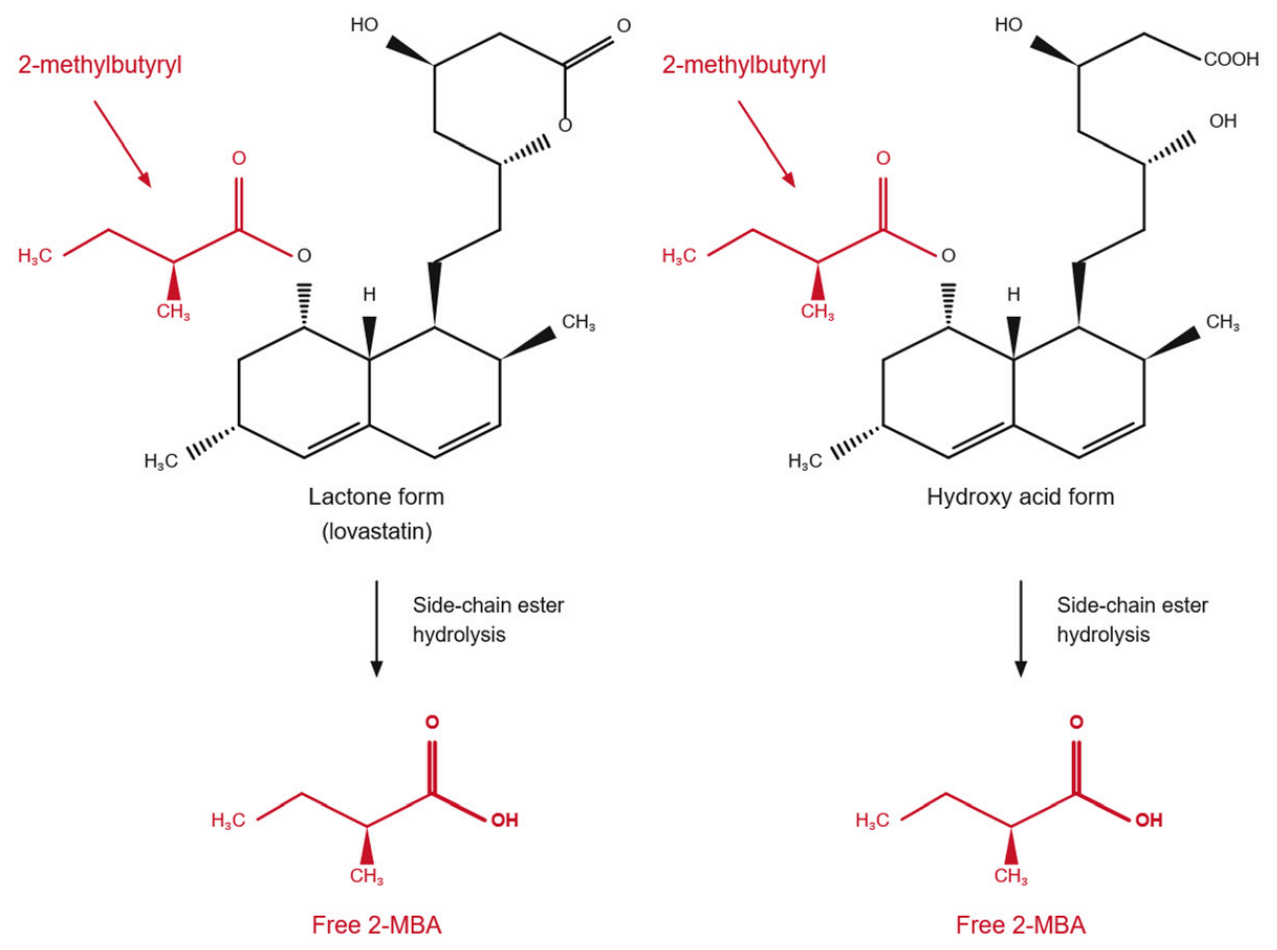
Monacolin K (MK) and lovastatin share a 2-methylbutyryl side chain, which is released as free 2-methylbutyric acid (2-MBA) upon hydrolysis of the side-chain ester bond (black arrows). MK is shown in its lactone (lovastatin) and hydroxy acid forms. The bound side chain and free 2-MBA are highlighted in red, with the carboxyl group of free 2-MBA shown in bold red. The corresponding deacylated products are omitted for clarity.

Red yeast rice (RYR), known as hongqu in China, is produced by fermenting steamed rice with *Monascus purpureus* [1,2]. Its cholesterol-lowering activity is attributed mainly to monacolin K (MK), which accounts for 75–90% of the total statin content in naturally fermented products [3,4]. MK competitively inhibits 3-hydroxy-3-methylglutaryl coenzyme A (HMG-CoA) reductase, thereby lowering serum cholesterol [5,6]. The compound occurs in two interconvertible forms, hydroxy acid and lactone (Figure 1); the lactone form is structurally identical to pharmaceutical lovastatin [7,8]. Both the lactone and hydroxy acid forms of MK have been determined in RYR products by chromatographic profiling [9,10]. This shared structure complicates authenticity control and regulatory monitoring because it prevents molecular identification alone from distinguishing endogenous MK from illegally added pharmaceutical lovastatin [11,12].

Native MK content varies substantially between RYR production batches, largely because fermentation conditions differ across production runs [4,11,13]. Pharmaceutical lovastatin may be added to raise the concentration to a product’s label claim; this risk of illicit spiking has been documented in international studies [14,15,16,17]. RYR is widely sold as a dietary supplement, so the undeclared addition of pharmaceutical lovastatin constitutes economically motivated food adulteration [12,13,14]. Such products can expose consumers to prescription-strength medication without their knowledge. Reliable methods for distinguishing the source of the statin are consequently needed for food and drug safety monitoring, alongside the methods already used to determine its concentration.

Chromatography, mass spectrometry, and nuclear magnetic resonance (NMR) can quantify monacolins in RYR [18]. These measurements do not, however, establish their source: MK lactone and pharmaceutical lovastatin have the same molecular structure [12,13,14]. Stable isotope ratio analysis addresses a different property, the isotope signature associated with production materials and processes [19,20]. Isotope studies of rice, the substrate for RYR fermentation, illustrate the relevance of substrate carbon to source discrimination [21,22]; microbial biosynthesis may also influence the resulting signature. Differences between MK isolated from RYR and commercial lovastatin have previously supported the use of δ^13^C analysis for authenticity screening [14,15].

Earlier isotope studies measured intact MK/lovastatin after chromatographic isolation [14,15]. Perini et al. [15] examined 18 RYR and 18 lovastatin samples using preparative HPLC and intact-molecule isotope analysis, with a reported screening boundary of −28.3‰. Hannon et al. [14] used HPLC collection, solid-phase extraction, and EA-IRMS; their minimum analyzable MK content was >0.8 mg/g under the reported preparation conditions, and interlaboratory differences for five powders were 0.02–0.76‰. Here, the target is a structurally defined hydrolysis product, 2-MBA, whose isotope composition represents a specific molecular fragment (Figure 1). Chromatographic separation remains necessary, since co-extracted carbon from complex commercial formulations can contribute to the measured isotope signal. This workflow avoids offline preparative isolation and collection of intact MK/lovastatin before isotope measurement, using liquid–liquid extraction followed by online separation of 2-MBA. Within-day repeatability was 0.19‰ for RYR, and interlaboratory SDs were 0.18–0.19‰ under the investigated conditions (Section 3.2.1). Fragment and intact-molecule isotope values represent different carbon pools, so their screening thresholds are not interchangeable.

Hydrolysis cleaves the ester bond in MK (lovastatin), releasing monacolin J and the 2-methylbutyryl side chain as 2-methylbutyric acid (2-MBA) [23,24]. The side chain has a distinct biosynthetic origin. In *Aspergillus terreus*, the dedicated polyketide synthase LovF synthesises the 2-methylbutyryl moiety, which is then transferred to the monacolin J scaffold during lovastatin biosynthesis [25,26,27]. Reviews describe the biosynthesis and metabolic regulation of monacolin K [28], while work in *Monascus* spp. has characterised a homologous pathway involving a dedicated enoyl reductase [29]. Hydrolysis retains the side chain’s carbon skeleton, allowing 2-MBA to be examined by fragment-specific stable carbon isotope analysis. The working hypothesis was that differences in the production origins of RYR-derived MK and pharmaceutical lovastatin would remain detectable in the δ^13^C values of this hydrolysis product.

In this study, an LC-IRMS method was developed for the fragment-specific determination of δ^13^C in hydrolysis-derived 2-MBA. Using fixed hydrolysis conditions with evaluation of extraction and isotope-measurement performance, the method was assessed in authentic RYR samples and pharmaceutical lovastatin products and subsequently applied to commercial RYR products; its isotopic response in an authentic RYR matrix was further examined using controlled blends with pharmaceutical lovastatin. The study aimed to assess whether the δ^13^C signature of 2-MBA could provide a targeted screening tool for identifying RYR products that are isotopically inconsistent with an authentic reference population.

## 2. Materials and Methods

### 2.1. Samples

The authentic RYR reference set comprised 45 samples from ten Chinese manufacturers: 34 raw materials and 11 finished products from Zhejiang, Guizhou, Shandong, Guangdong, Hubei, Beijing, Sichuan and Shanxi. Manufacturers are identified as Suppliers A–J in Table S1. Another 20 commercial RYR products, including capsules, tablets and powders, were purchased at retail; 16 carried numerical MK declarations (Table S1).

Twenty-three pharmaceutical lovastatin batches, supplied as capsules or tablets, were purchased from pharmacies in Anhui, Jiangsu, Shandong, Sichuan and Henan (Table 1 and Table S1). RYR products and pharmaceutical lovastatin products were stored at room temperature in a dry place and protected from light until analysis. Lovastatin and 2-MBA working standards were stored in a refrigerator at 4–8 °C. Samples were kept in their original sealed packaging or, after subsampling, in sealed polyethylene containers. Storage duration was not recorded.

### 2.2. Reagents and Reference Materials

Five lovastatin working standards (LRS; purity ≥ 98%; CAS 75330-75-5) and two 2-MBA working standards (purity ≥ 98%; CAS 116-53-0) were obtained from commercial suppliers in Shanghai and Beijing for calibration and isotope validation. Supplier details are listed in Table 1.

All solvents and reagents were of analytical grade or higher purity unless otherwise specified. Helium and carbon dioxide (99.999%) for LC-IRMS analysis were supplied by Air Products and Chemicals, Inc. (Beijing, China). Sodium persulfate (Na_2_S_2_O_8_, ≥99%) and methanol (CH_3_OH, HPLC grade) were purchased from Sinopharm Chemical Reagent Co., Ltd. (Shanghai, China). Ultrapure water (18.2 MΩ·cm) was produced using a Milli-Q water purification system (Millipore, Bedford, MA, USA). The isotope reference material IAEA-CH-6 (sucrose; assigned δ^13^C = −10.45‰; standard uncertainty = 0.03‰, k = 1) was obtained from the International Atomic Energy Agency (IAEA, Vienna, Austria).

### 2.3. Sample Pretreatment

The hydrolysis strategy was based on the established cleavage of the 2-methylbutyryl ester moiety of lovastatin. Previous studies have shown that hydrolysis of lovastatin releases the 2-methylbutyryl side chain as 2-methylbutyric acid [24,30,31]. Ester bonds are generally more labile than amide bonds under acid-catalysed hydrolysis [32]. On this basis, combined with the compositional characteristics of red yeast rice and referring to the Chinese national standard (GB 5009.124—2016) [33], the hydrolysis conditions of 2-MBA for LC-IRMS analysis were used here as fixed operating settings informed by this chemical basis rather than established by systematic temperature–time optimisation. RYR samples were ground to a powder when necessary. Approximately 5.0 g was placed in a 50-mL centrifuge tube with 30 mL of 6 mol/L hydrochloric acid, ultrasonicated and vortexed to disperse the sample, and hydrolysed in the capped tube at 65 °C for 12 h. After cooling, 10 mL of diethyl ether was added, followed by vortexing for 30 s and centrifugation at 5000 r/min for 6 min. At the acidity of the hydrolysate, 2-MBA (pKa ≈ 4.8 in water at 25 °C [34]) is expected to remain predominantly non-ionised and to partition into the ether phase. The upper organic phase was transferred to another 50-mL tube. Extraction was repeated once, and the two organic phases were combined. The extract was mixed with 2 mL of potassium hydroxide in methanol (2 g/100 mL) and vortexed for 2 min. Using 2 mol/L aqueous sulfuric acid, the ether–methanol mixture was adjusted to approximately pH 8, as judged with broad-range pH indicator paper; this was an operational reading in the extraction mixture. Diethyl ether and methanol were evaporated at 50 °C under a gentle nitrogen stream. The residue was mixed with 3 mL of water and dried at 105 °C, reconstituted with 1 mL of water and dried again at 105 °C, then reconstituted with a further 1 mL of water. After centrifugation at 10,000 r/min for 10 min, the supernatant was collected and mixed with 50 μL of 2 mol/L sulfuric acid to prepare the pre-injection stock solution. A 200-μL aliquot was diluted with 900 μL of water, vortexed, passed through a 0.22-μm hydrophilic membrane filter and stored until analysis. The GC procedure for 2-MBA recovery assessment was based on the Chinese national standard (GB 28336—2012) [35] (Supplementary Section S2).

### 2.4. LC-IRMS Analysis

Hydrolysis-derived 2-MBA was analysed using an UltiMate 3000 liquid chromatograph, an IsoLink II wet oxidation interface and a Delta V Advantage isotope ratio mass spectrometer (Thermo Fisher Scientific, Bremen, Germany). Wet-oxidation LC-IRMS and its use in food authenticity testing are described elsewhere [36,37]. Coupling of liquid chromatography with ^13^C isotopic analysis has been reviewed more broadly [38]. Separation used a HyperREZ XP Carbohydrate H+ column (300 × 7.7 mm, 8 μm) at 30 °C. The mobile phase was ultrapure water containing 0.05 M sulfuric acid, delivered at 600 μL/min, and the injection volume was 10 μL.

The separated analyte entered the wet oxidation interface, where 0.25 mol/L sodium persulfate solution and 0.5 mol/L phosphoric acid solution were each supplied continuously at 150 μL/min. The reactor was held at 99.5 °C to convert analyte carbon to CO_2_. Helium carried the resulting CO_2_ to the IRMS for measurement of the ^13^C/^12^C ratio, following the isotope-ratio-monitoring principle described for this interface [39].

### 2.5. EA-IRMS Analysis

Owing to the limited aqueous solubility of lovastatin and the restriction on carbon-containing organic solvents in LC-IRMS, EA-IRMS was used to determine the δ^13^C values of the intact lovastatin working standards. This determination served solely to verify the isotopic relationship between intact lovastatin and its hydrolysis-derived 2-MBA (Section 3.2.2), rather than as a routine sample measurement. Hydrolysis-derived 2-MBA, which was compatible with the aqueous LC-IRMS system, was analysed by LC-IRMS. A 0.1–0.2 mg portion was weighed into a tin capsule (Thermo Fisher Scientific, Bremen, Germany) and analysed with a Flash 2000 elemental analyser coupled through a Conflo IV interface to a Delta V Advantage isotope ratio mass spectrometer (Thermo Fisher Scientific, Bremen, Germany).

Samples were combusted at 980 °C and subsequently reduced at 680 °C to ensure complete conversion of carbon into CO_2_. Helium was used as carrier gas at 100 mL/min, and oxygen was introduced for 3 s at 250 mL/min to support complete combustion. The separation column was maintained at 60 °C throughout the analysis.

### 2.6. Calibration and Data Analysis

Carbon isotope delta values (δ^13^C) are reported on the VPDB-LSVEC scale, anchored to the assigned value of IAEA-CH-6. Equation (1) defines the isotope delta notation used here:

(1) $$\delta^{13}C=\left[\frac{R_{\mathrm{sample}}}{R_{\mathrm{standard}}}-1\right]\times 1000$$

where *R_sample_* is the sample ^13^C/^12^C ratio and *R_standard_* is the ratio of the isotope-scale reference, rather than that of a working standard.

IAEA-CH-6 (δ^13^C = −10.45‰; standard uncertainty u = 0.03‰, k = 1, on the VPDB-LSVEC scale) was used as the isotope reference material for calibration [40]. The δ^13^C values of the lovastatin and 2-MBA working standards were calibrated against IAEA-CH-6 using EA-IRMS and LC-IRMS, respectively, and the calibrated working standards were subsequently used for routine sample measurements and quality control. All samples were analysed in triplicate, and the mean δ^13^C value was used for subsequent data analysis.

### 2.7. Method Validation

Validation covered repeatability, within-laboratory and interlaboratory reproducibility, mixture accuracy and measurement uncertainty [41,42,43]. Pretreatment-related isotope effects were evaluated by comparing the δ^13^C values of two 2-MBA working standards before and after the complete pretreatment procedure.

### 2.8. Statistical Analysis

Descriptive statistics were calculated for all δ^13^C datasets. For reference-population and commercial-product analyses, the production batch was the sampling unit: triplicate analytical measurements were averaged to obtain one δ^13^C value per batch and were not counted as additional samples. The two reference populations were assessed for normality using the Shapiro–Wilk test and Q–Q plots. One-sided parametric upper tolerance limits (UTLs) were calculated for the authentic RYR population at 95% confidence and 90%, 95% and 99% population coverage; the 99% coverage limit was selected as the investigational screening threshold. One-sided tolerance factors for the normal distribution are tabulated in ISO 16269-6 [44]; the underlying theory is set out by Meeker et al. [45], and the sample sizes needed to support such limits are discussed by Kirkpatrick [46]. Because the upper-tail estimate depends on the reference population and normal-model assumptions, the threshold is population-specific. Full calculations and sensitivity analyses are given in Supplementary Section S4.

(2) $$\mathrm{UTL}=\bar{x}+k\cdot s$$

(3) $$k=\frac{t\left(1-\alpha ;n-1;z_{p}\sqrt{n}\right)}{\sqrt{n}}$$

Here, $\bar{x}$ and *s* are the sample mean and sample standard deviation (denominator *n* − 1), and *k* is the one-sided normal tolerance factor. For confidence level 1 − *α* and population coverage p, *k* is obtained from a non-central t distribution with *n* − 1 degrees of freedom and non-centrality parameter $z_{p}\sqrt{n}$, divided by $\sqrt{n}$; *z_p_* is the standard-normal quantile for p. For *n* = 45 and 95% confidence, *k* is 1.669, 2.092 and 2.898 at 90%, 95% and 99% coverage, respectively. Tolerance intervals of this form are widely used to set specification limits for normally distributed attributes [47]. Calculations were performed in Python 3.13 with NumPy and SciPy; the complete derivation is provided in Supplementary Section S4 and Table S6.

## 3. Results and Discussion

### 3.1. Development of the Hydrolysis–Extraction Workflow

**Figure 2:**
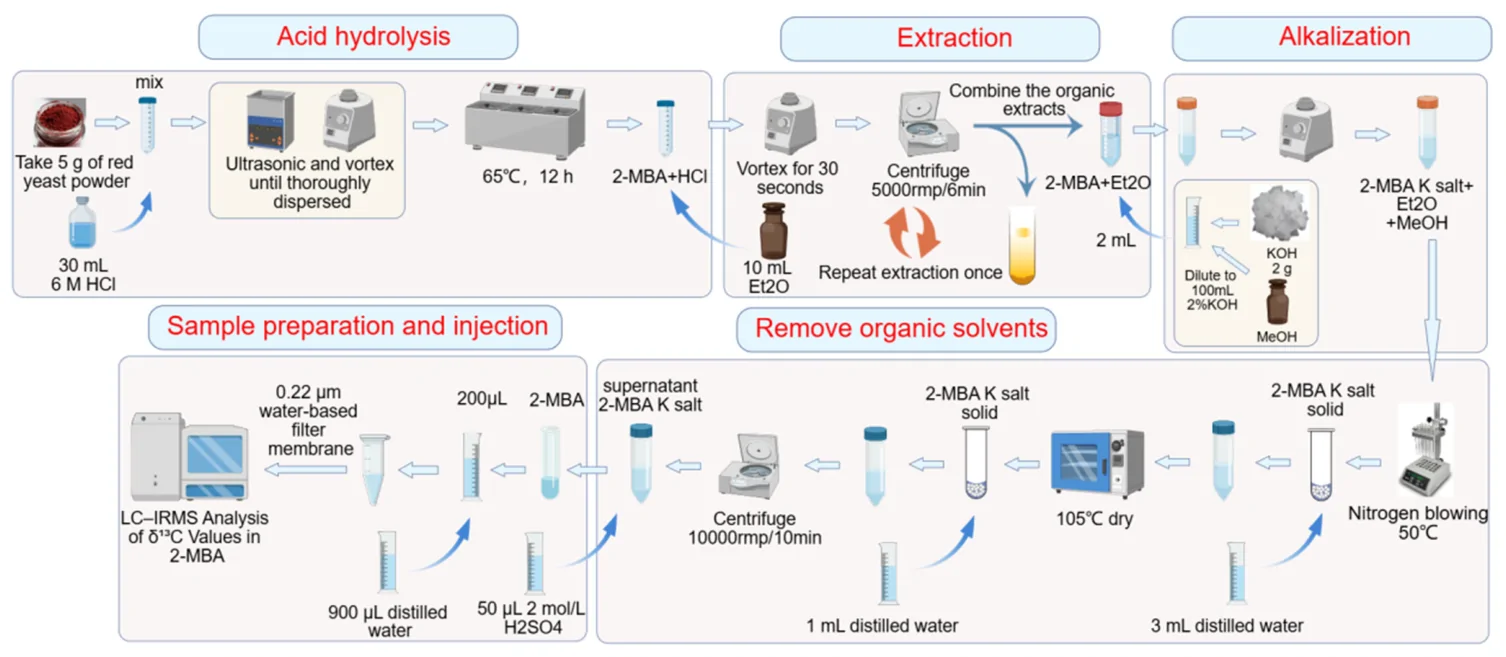
RYR sample pretreatment workflow. Before evaporation, the ether–methanol extract was adjusted to an operational pH of approximately 8 with 2 mol/L aqueous sulfuric acid. After the two aqueous dissolution–drying cycles, the residue was reconstituted in a further 1 mL of water before centrifugation and preparation of the injection solution (Section 2.3).

The workflow was developed to recover 2-MBA as a chromatographically resolved peak for isotope analysis (Figure 2). Solvent choice, extraction frequency and solvent removal were examined, while the hydrolysis conditions remained fixed as described in Section 2.3. Evidence concerning pretreatment effects on isotope measurements and its limitations are discussed in Section 3.2.2.

Following hydrolysis with 6 mol/L HCl at 65 °C for 12 h, the reported recovery estimates were higher for diethyl ether than for MTBE or n-hexane (Table S2). Two successive ether extractions gave a mean reported recovery estimate of 97.6% (RSD, 2.5%; *n* = 3); a third extraction provided no material gain (Table S3). Residual ether was not detected by GC after the second dissolution–drying step at 105 °C (Figure S1). Reported recovery was calculated from the GC-derived amount in the combined organic extracts relative to the theoretical 2-MBA yield from lovastatin (Supplementary Section S2.3). Solvent selection followed conventional liquid–liquid extraction practice [48]. Acid treatment may also denature protein and disrupt part of the starch–protein matrix, reducing entrapment of MK [49]. Extraction conditions reported for optimised MK recovery from RYR [50] differ from those used here, so the corresponding recovery values are not directly transferable.

**Figure 3:**
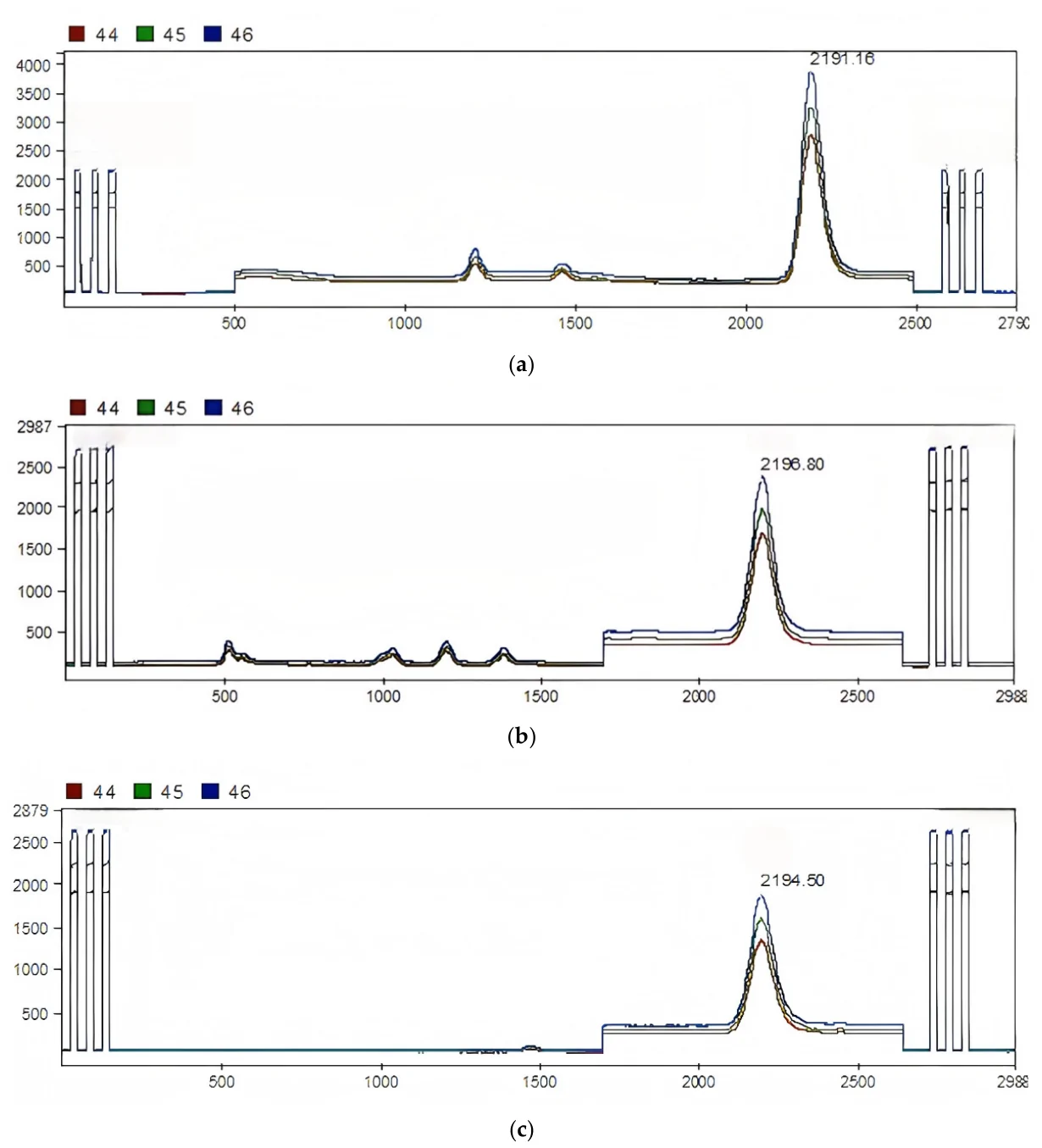
LC-IRMS chromatograms of 2-MBA: (**a**) standard, (**b**) lovastatin hydrolysate and (**c**) RYR hydrolysate. Channels 44, 45 and 46 denote CO_2_ ion signals at *m*/*z* 44, 45 and 46, respectively.

Chromatographic selectivity was examined using a 2-MBA working standard and hydrolysates of lovastatin and RYR. The 2-MBA standard and the target peaks in both hydrolysates had consistent retention times under the same LC-IRMS conditions (Figure 3), with no separate interfering peak evident in the 2-MBA region. This agreement supports assignment of the target peak and its separation from the observed matrix peaks.

### 3.2. Analytical Performance of the LC-IRMS Method

#### 3.2.1. Precision and Reproducibility

Table 2 and Table 3 summarise instrument stability and analytical precision for 2-MBA δ^13^C measurements. Ten consecutive CO_2_ injections gave an SD of 0.026‰. Six independent replicate measurements gave within-day repeatability of 0.19‰ for RYR and 0.05‰ for pharmaceutical lovastatin. Within-laboratory reproducibility was assessed from ten measurements over three consecutive days, yielding an SD of 0.29 ‰. Each series was evaluated separately because it measured a different aspect of precision.

The standard and two market-collected RYR materials gave interlaboratory SDs of 0.18–0.19‰ across three laboratories (Table 4). RYR Sample 1 had a mean of −26.32‰, above the −26.47‰ screening threshold (Section 3.3.2). The comparison tested agreement among laboratories; it did not establish the authenticity of the market materials, for which adulteration could not be excluded. Sample 1 therefore warrants further investigation. RYR Sample 1 and RYR Sample 2, the two market-collected materials used in the inter-laboratory comparison for which adulteration could not be excluded, are not listed in Table 1 or Table S1, because they were procured for the inter-laboratory comparison rather than as part of the investigated reference or commercial sample sets.

#### 3.2.2. Pretreatment Effects on Isotope Measurements

Chemical processing can cause isotope fractionation and therefore requires explicit assessment. Two 2-MBA working standards were measured before and after the complete pretreatment procedure. Their δ^13^C values changed from −8.95‰ to −8.78‰ and from −23.59‰ to −23.65‰, corresponding to deviations of −0.17‰ and +0.06‰, respectively, where the deviation is defined as the value before minus that after the complete pretreatment procedure (Table S9). These small changes indicate that no substantial isotope shift was observed for the two tested 2-MBA standards under these pretreatment conditions.

**Figure 4:**
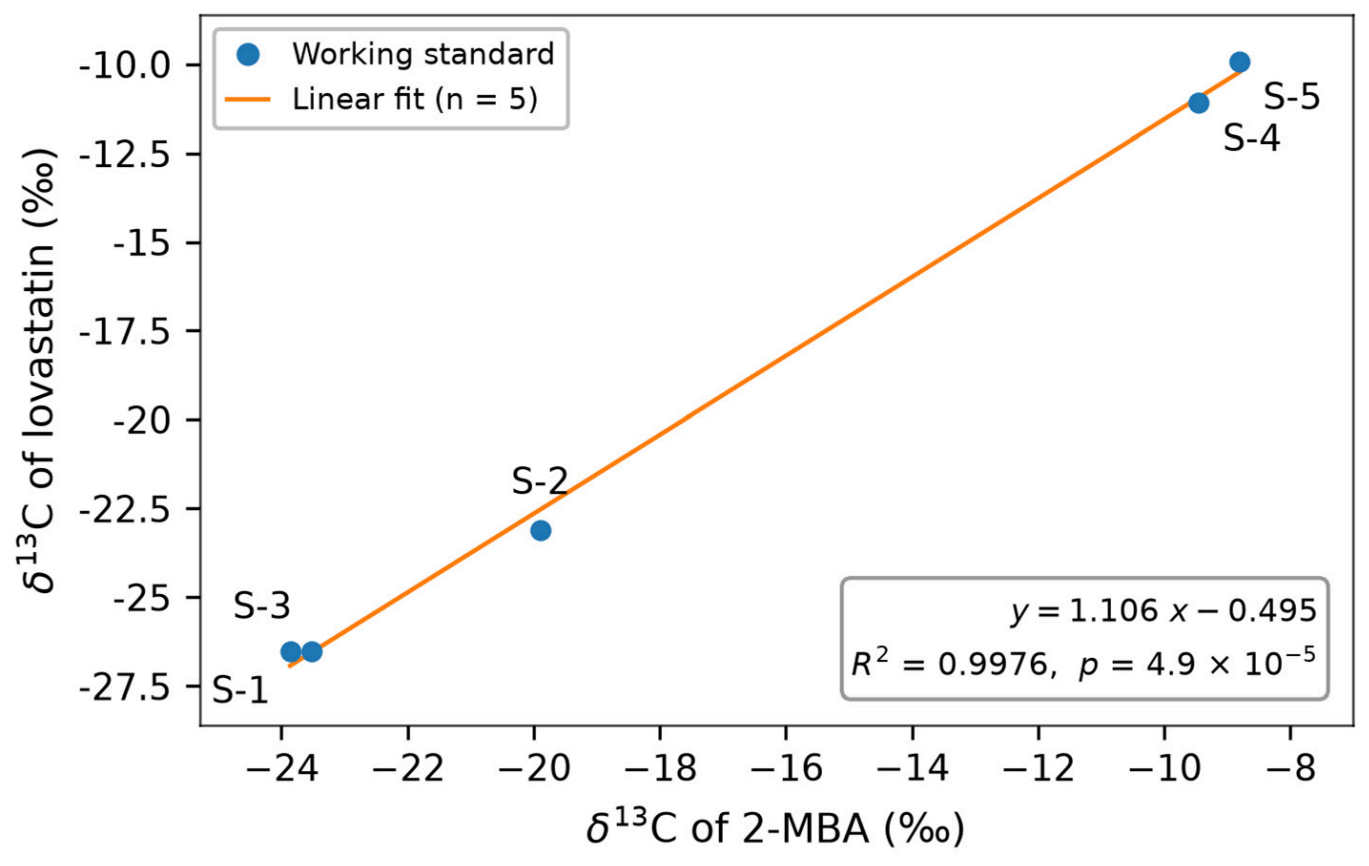
Paired δ^13^C values for the same five lovastatin working standards, measured intact by EA-IRMS and as hydrolysis-derived 2-MBA by LC-IRMS.

The same five lovastatin working standards were measured intact by EA-IRMS and as hydrolysis-derived 2-MBA by LC-IRMS. The resulting δ^13^C values were strongly correlated (R^2^ = 0.9976, ***p*** = 4.9 × 10^−5^; Figure 4), indicating that the relative isotopic differences among the standards were retained in the 2-MBA fragment. Because intact lovastatin and its 2-methylbutyryl fragment represent different carbon pools, this correlation does not directly quantify isotope fractionation specifically associated with hydrolytic cleavage.

#### 3.2.3. Agreement with Mass-Balance Predictions for Laboratory-Prepared Mixtures

**Figure 5:**
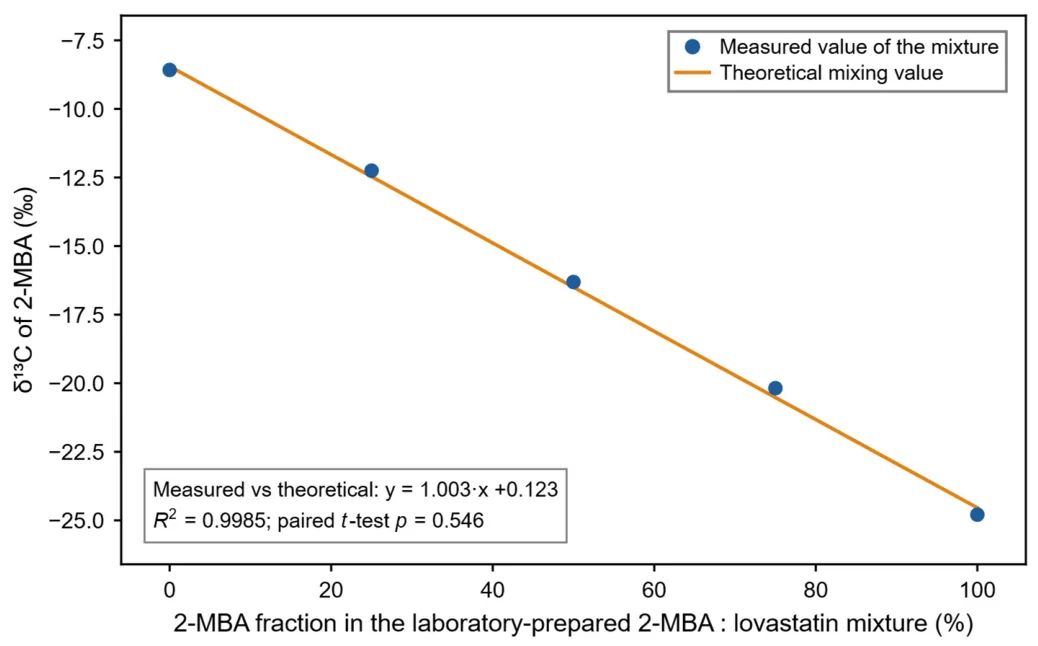
Measured and predicted δ^13^C values for mixtures of lovastatin and 2-MBA working standards. The *x*-axis represents the 2-MBA molar fraction (%), not a mass fraction. Predictions use the assigned endmember values of −8.45‰ and −24.54‰, respectively (Table S4); the correlation and paired test assess agreement with this specified mixing model.

Five mixtures of 2-MBA and lovastatin working standards were analysed in duplicate. Measured δ^13^C values differed from mass-balance predictions by 0.13–0.34‰ in absolute terms (Table S4; Figure 5; R^2^ = 0.9985). A paired comparison of measured and predicted values found no statistically significant mean residual (*p* = 0.55). A non-significant paired test does not demonstrate equivalence or accuracy against an independent certified 2-MBA value.

#### 3.2.4. Measurement Uncertainty

A two-component uncertainty estimate was calculated following JCGM 100:2008 (GUM) [51]. The ten within-laboratory results in Table 3 gave a sample SD of 0.28797‰, used as the Type A reproducibility component. Combining this with the IAEA-CH-6 assigned-value uncertainty of 0.03‰ (Type B), assuming independence and unit sensitivity coefficients, gave a combined standard uncertainty of 0.28953‰. Using a coverage factor k = 2 gave an expanded uncertainty of 0.57906‰, reported as ±0.58‰. The full reproducibility component was retained for sample means because triplicate averaging does not necessarily reduce shared calibration or between-day effects.

Only reproducibility and reference-value uncertainty are included in this estimate. Working-standard assignment and calibration transfer, isotope-scale nonlinearity, pretreatment bias and matrix-dependent contributions were not quantified separately. The result is a two-component estimate rather than a complete uncertainty budget for every sample matrix. This distinction matters when interpreting results near the screening threshold (Section 3.5).

### 3.3. Isotopic Screening of RYR Products Based on the δ^13^C Analysis of 2-MBA

#### 3.3.1. Isotopic Discrimination Between Authentic RYR and Pharmaceutical Lovastatin

**Figure 6:**
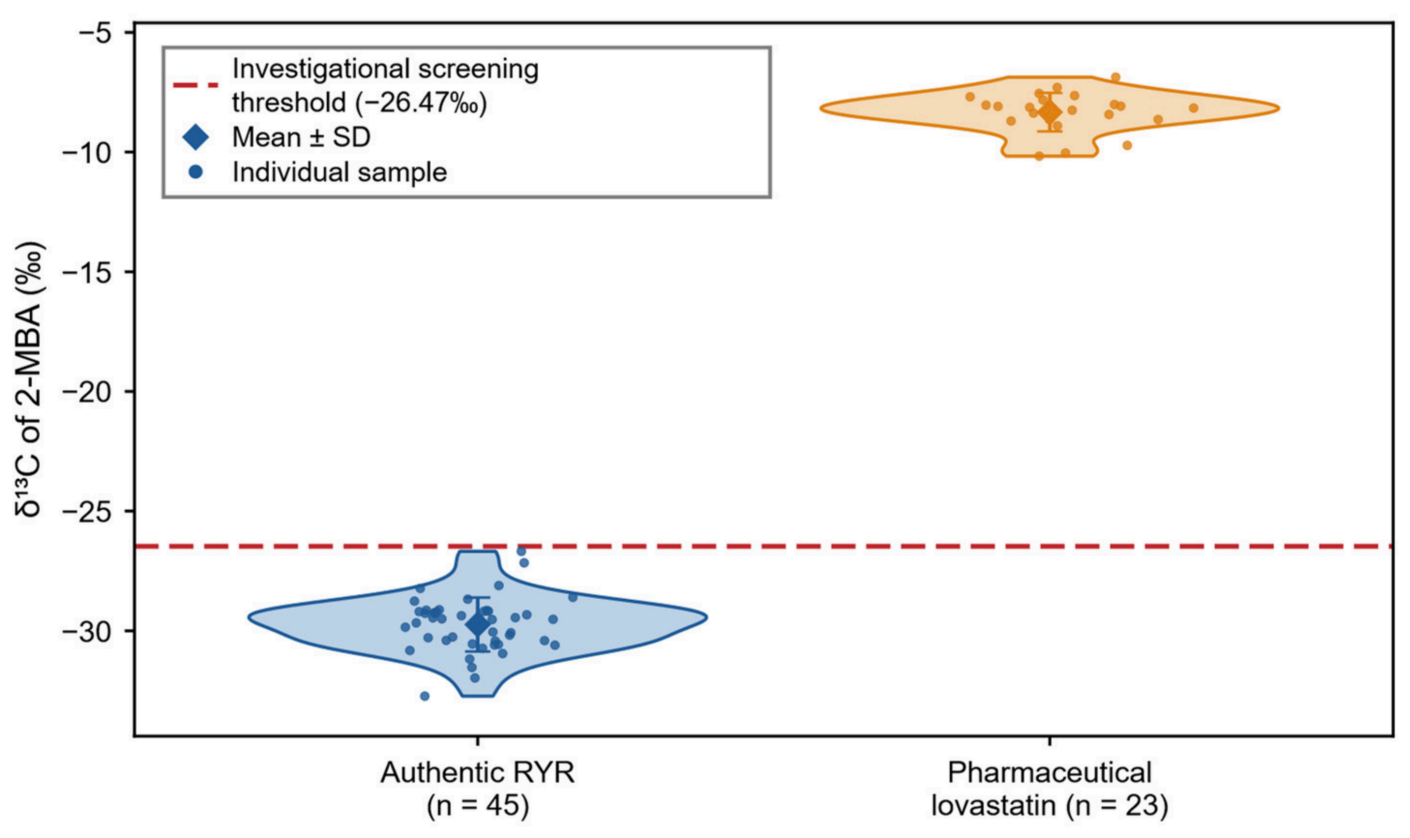
δ^13^C distributions of 2-methylbutyric acid (2-MBA) in the reference populations: authentic RYR (blue, *n* = 45) and pharmaceutical lovastatin (orange, *n* = 23). Violin plots show the kernel density distribution, with individual samples overlaid as dots and the mean ± standard deviation indicated by diamonds. The red dashed line denotes the population-specific screening threshold of −26.47‰ (see Section 3.3.2).

Hydrolysis-derived 2-MBA ranged from −32.73‰ to −26.68‰ in the 45 authentic RYR samples (mean ± SD, −29.74 ± 1.13‰) and from −10.17‰ to −6.87‰ in the 23 pharmaceutical lovastatin batches (−8.33 ± 0.80‰) (Table 5; Figure 6). The sampled batches showed no overlap. These ranges characterise the investigated products and should not be treated as universal values for either population.

#### 3.3.2. Selection of a Population-Specific Screening Threshold

The 45 authentic RYR samples from ten Chinese manufacturers showed no marked departure from normality (Shapiro–Wilk W = 0.969, *p* = 0.260; excess kurtosis +1.23; Figure S2; Table S5), and no Grubbs outliers were detected. Neither check establishes normality. With *n* = 45, a distribution-free upper limit cannot attain 99% coverage at 95% confidence. The selected boundary therefore uses a one-sided parametric UTL and the sample standard deviation (*n* − 1), with the model assumptions and derivation set out in Supplementary Section S4.

At 95% confidence, the UTLs for 90%, 95% and 99% population coverage were −27.86‰, −27.38‰ and −26.47‰, respectively (Table 6). The 99% coverage limit was selected as the investigational screening threshold. Its 95% bootstrap interval, −27.41‰ to −25.62‰, illustrates the uncertainty of the upper-tail estimate (Table S7). Under the fitted normal model, the nominal probability of exceeding this limit is 0.19%. This is below 1%, the complement of the selected coverage, because the limit also accommodates the 95% confidence requirement. It is not an experimentally determined false-positive rate for future products. The −26.47‰ boundary is specific to the investigated Chinese reference population; an exceedance signals isotopic inconsistency with that population and requires further evidence before adulteration can be established. Omitting the highest observation (R41, −26.68‰), the second-highest observation (R29, −27.16‰), or both shifted the limit to −26.79‰, −26.69‰ and −27.05‰, respectively, and the 95% chi-square confidence interval of the sample standard deviation was 0.933–1.424‰ (Table S7), indicating that the threshold is sensitive to the upper tail of the present reference set and that results close to the boundary should be interpreted with caution.

### 3.4. Controlled Blending of Authentic RYR with Pharmaceutical Lovastatin

**Figure 7:**
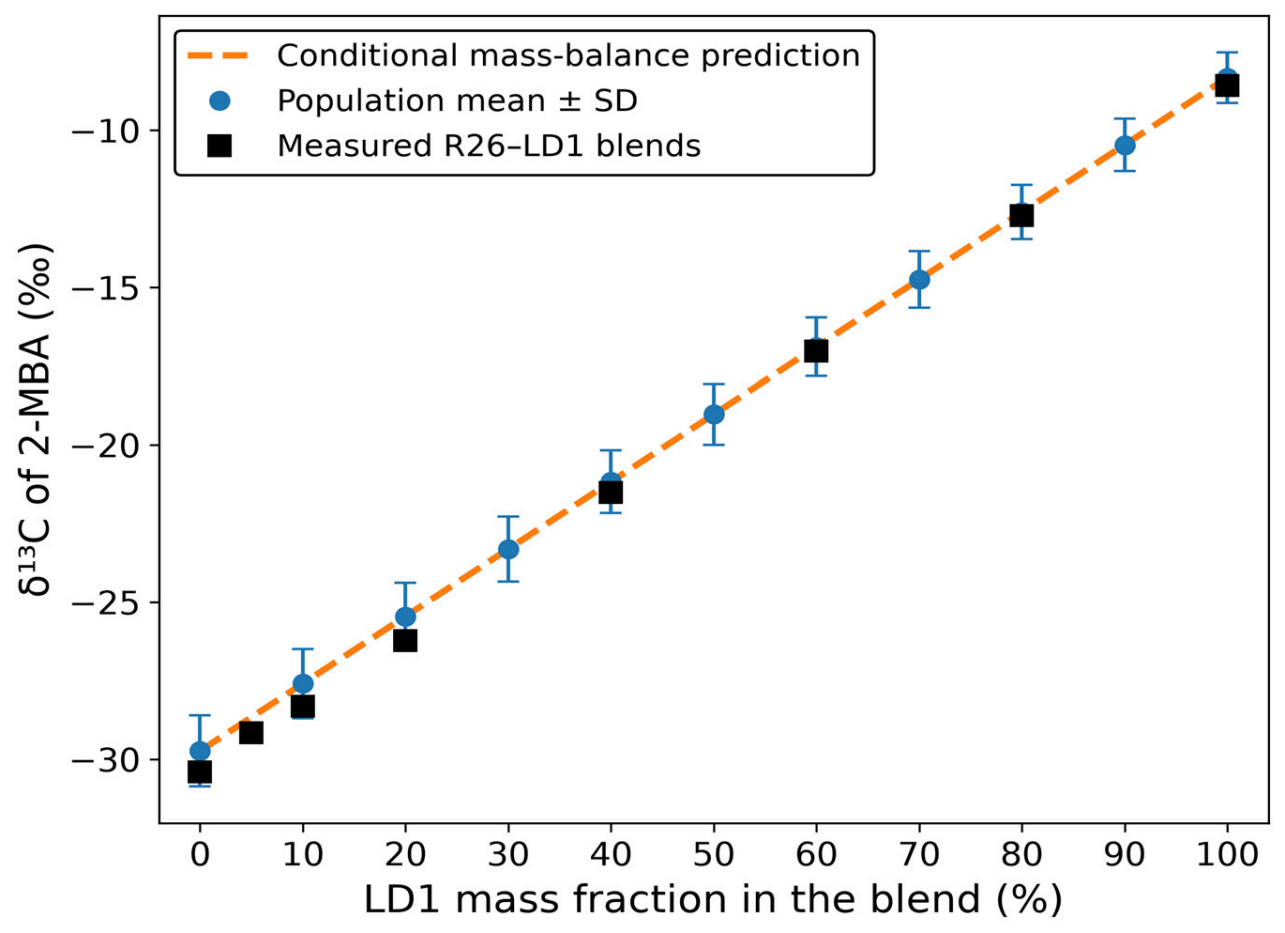
Measured δ^13^C values for all eight R26–LD1 blends (black squares; Table S8) and the conditional mass-balance prediction (dashed line). The model uses R26-derived 2-MBA at −30.41‰ and the assigned lovastatin input at −8.45‰; the measured LD1 endpoint is −8.58‰. The *x*-axis is LD1 mass fraction in the blend, not pure lovastatin mass percentage. Interpreting this fraction as a side-chain contribution requires the assumptions in Section 3.4. The horizontal line is the investigational screening threshold of −26.47‰.

To evaluate the isotope response in an authentic RYR matrix, a conditional mixing model was considered using the hydrolysis-derived 2-MBA distributions of authentic RYR (−29.74 ± 1.13‰, *n* = 45) and pharmaceutical lovastatin (−8.33 ± 0.80‰, *n* = 23). These are fragment-specific values, not intact-MK/lovastatin values. Controlled blends were prepared from authentic RYR sample R26 (measured MK content, 5.22%; hydrolysis-derived 2-MBA δ^13^C, −30.41‰; content determined according to QB/T 2847-2023 [52]) and lovastatin–dextrin powder LD1 (nominal lovastatin content, 5.22%). RYR:LD1 mass ratios were 100:0, 95:5, 90:10, 80:20, 60:40, 40:60, 20:80 and 0:100. Table S8 predictions use −8.45‰ as the assigned lovastatin working-standard/model input, whereas the 0:100 LD1 preparation measured −8.58‰ in the current analytical sequence. All eight blends underwent the complete pretreatment and LC-IRMS procedure. Measured δ^13^C values followed a linear response (R^2^ = 0.9997, *n* = 8 blending levels); absolute differences from the stated model predictions were 0.11–0.21‰ for the seven levels with numerical predictions in Table S8 (Figure 7). The agreement supports the response of this tested R26–LD1 pair, without establishing performance across other matrices or sources.

By isotope mass balance, the −26.47‰ threshold would be crossed when lovastatin contributes approximately 15% of the hydrolysable 2-methylbutyryl pool for the mean end-member values, and approximately 18% for the R26–LD1 pair. These estimates assume that both components contribute 2-MBA in proportion to their nominal mixing ratio; they depend on the δ^13^C value of the authentic matrix, the lovastatin source and the relative analyte contents. For the R26–LD1 pair, the modelled crossing corresponds to approximately 0.94% (*w*/*w*) exogenous lovastatin in the final blend (Supplementary Section S4). These are model-based threshold crossings, not experimentally established minimum detectable adulteration levels across matrices. Equating the LD1 mass fraction with its contribution to the hydrolysable 2-methylbutyryl pool additionally assumes comparable molar side-chain yields from R26 and LD1; equal nominal mass contents alone do not verify this assumption, particularly when MK acid and lactone forms are combined.

### 3.5. Application to Commercial RYR Products

**Figure 8:**
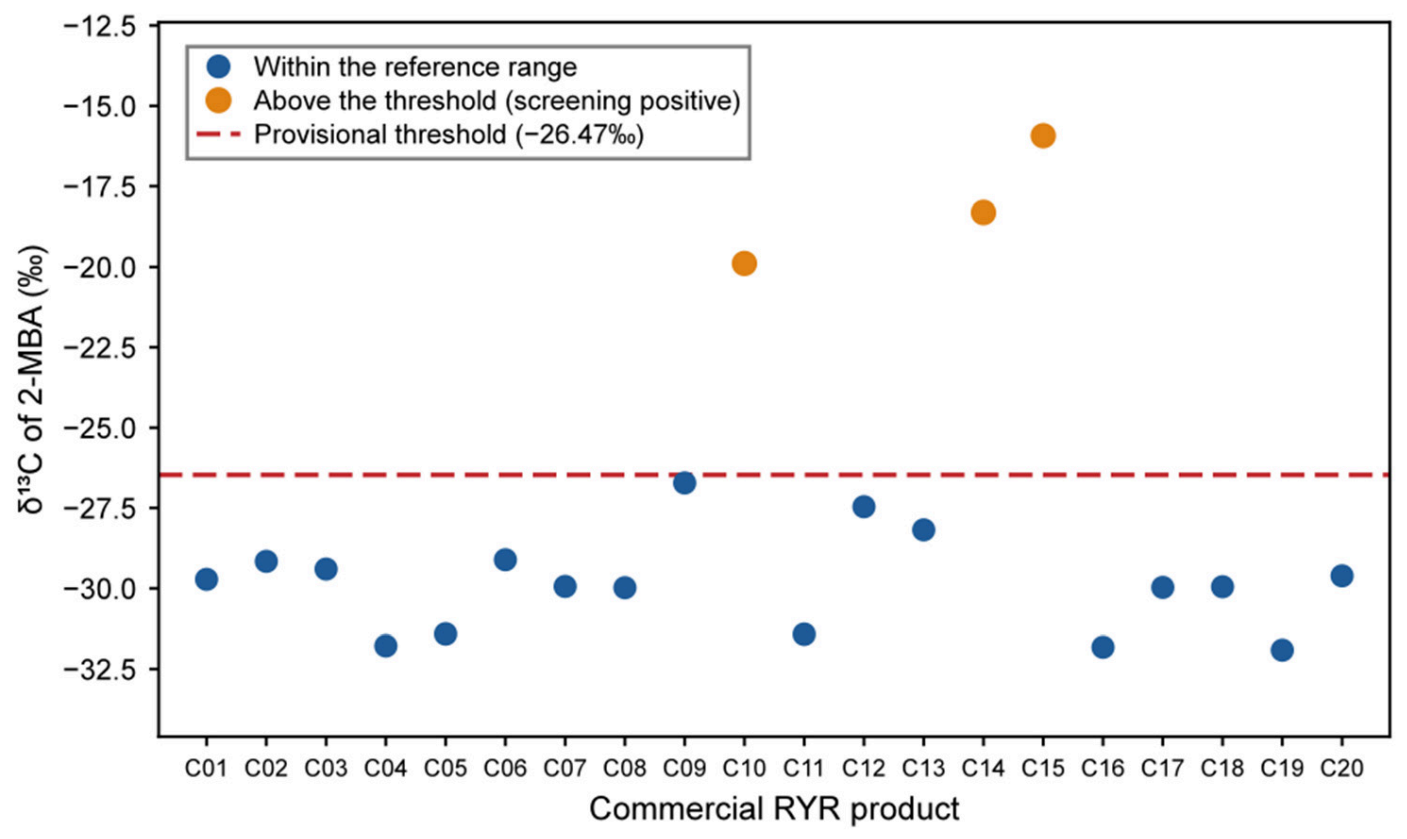
Hydrolysis-derived 2-MBA δ^13^C values for all 20 commercial RYR products (Table S1). Blue circles indicate values below the investigational threshold; orange circles indicate screening positives relative to the sampled Chinese authentic RYR population. Neither category establishes authenticity or adulteration. C09 (−26.72‰) is close to the −26.47‰ boundary relative to the two-component expanded uncertainty estimate and requires additional evidence. No replicate error bars are shown.

Seventeen of the 20 commercial RYR products fell below the screening threshold (Figure 8; Table S1). C09 was only 0.25‰ below it, within the two-component expanded uncertainty estimate, and requires additional evidence for classification. C10, C14 and C15 exceeded the threshold, with δ^13^C values of −19.89‰, −18.31‰ and −15.93‰, respectively. They were classified as screening positives relative to the investigated Chinese reference population. No independent source-sensitive confirmation was performed, so these values do not establish pharmaceutical lovastatin addition. Monacolin quantification can assess compliance with a content claim; meeting that claim does not identify the statin’s origin.

Declared MK contents were not independently verified. Among the 16 products carrying numerical claims, the association between declared content and δ^13^C was not statistically significant (Spearman’s ρ = −0.46, *p* = 0.072). Excluding the three screening positives gave ρ = −0.19 (*p* = 0.54; Supplementary Section S4).

A concentration–response test was also performed: 2-MBA was added at 0.5%, 2.5%, 5.0% and 7.0% (*w*/*w*) to a single pigmented RYR material used for food colourant purposes, in which 2-MBA was not detected under the GC screening conditions, giving signal intensities of 1209–3342 mV and δ^13^C values from −8.72‰ to −8.55‰ (mean −8.62‰; SD across four concentration levels, 0.07‰; *n* = 4 levels). No concentration-dependent shift was apparent within the tested range (Supplementary Section S7, Table S10). This single-matrix test does not establish applicability at lower analyte contents or in other matrices.

### 3.6. Interpretation of the Isotopic Offset

The reference populations differed in mean δ^13^C by approximately 21‰, an offset compatible with differences in feedstock carbon. Rice, the substrate for *Monascus* fermentation, is a C3 plant. Terrestrial C3 plants commonly fall between −35‰ and −20‰, while C4 plants occupy roughly −19‰ to −9‰ [53]. The RYR values lie within the C3 interval; pharmaceutical values lie near, and partly above, the C4 interval (Table 5). Mixed or industrially processed carbon sources may also contribute. Neither fermentation substrates nor manufacturing records were available, so this interpretation is an inference rather than a documented attribution of production origin. Pretreatment effects are considered in Section 3.2.2, and isotope fractionation during microbial biosynthesis was not quantified.

### 3.7. Scope and Further Validation

The reference set covered ten Chinese manufacturers but was geographically uneven: Zhejiang supplied 25 of the 45 authentic RYR samples (56%), and two manufacturers there supplied 9 of the 11 finished products. Manufacturer and province analyses did not detect statistically significant group differences (F(9,35) = 0.79, *p* = 0.63; F(7,37) = 0.90, *p* = 0.52), with estimated ICC ≈ 0. Leave-one-group-out UTLs ranged from −26.58 to −26.25‰ for provinces and −26.74 to −26.07‰ for manufacturers (Table S11). Samples represented distinct batches manufactured between November 2024 and April 2026, although shared manufacturing processes may still induce dependence [54]. The pharmaceutical set comprised 23 batches, and controlled blending involved only one RYR matrix and one lovastatin source. Retention-time agreement between the 2-MBA standard and the lovastatin and RYR hydrolysates supports peak assignment (Figure 3), but does not distinguish free 2-MBA from other compounds releasing the same acid. Broader application requires geographically wider sampling, a balanced distribution of batches and manufacturers, independent threshold verification, and controlled blends spanning multiple matrices and sources. Matrix-specific marker contributions and source-sensitive confirmation also need assessment. Additional independent isotope reference materials spanning the sample range would be required to examine scale normalization beyond the single point used here.

## 4. Conclusions

The fragment-specific LC-IRMS workflow measured δ^13^C in hydrolysis-derived 2-MBA from RYR products. Precision, agreement in laboratory-prepared mixtures and interlaboratory comparisons support its analytical use under the investigated conditions. Authentic RYR and pharmaceutical lovastatin samples had distinct isotope distributions, and controlled blends followed the expected mixing response for the tested endmembers.

The −26.47‰ boundary provides an investigational screen for the Chinese reference population examined here. Exceedance denotes isotopic inconsistency and does not confirm adulteration. Independent validation across further reference populations, matrices and lovastatin sources is needed before the threshold can support broader use.

## Figures and Tables

**Table 1:** Study sample groups.

Category	Sample Type	Sample Size	Sample Source	Remarks
Authentic samples	RYR	45	Sourced from ten RYR manufacturers in China.	MK content reported: 27 (26 label claims; one measured value)
Commercial samples	Pharmaceutical lovastatin	23	Purchased from pharmacies	Capsules/tablets
	Commercial RYR products	20	Obtained from Chinese online retail platforms	Blind samples
Reference materials	lovastatin working standards (LRS)	5	L1: Shanghai Aladdin Biochemical Technology Co., Ltd. (Shanghai, China) L2: Shanghai Macklin Biochemical Co., Ltd. (Shanghai, China) L3: Beijing InnoChem Science & Technology Co., Ltd. (Beijing, China) L4: Shanghai Yuanye Bio-Technology Co., Ltd. (Shanghai, China) L5: Shanghai Haohong Scientific Co., Ltd. (Shanghai, China)	Standard substance for content analysis (purity ≥ 98%). CAS No. 75330-75-5
	2-MBA working standards	2	M1: Shanghai Aladdin Biochemical Technology Co., Ltd. (Shanghai, China) M2: Shanghai Macklin Biochemical Co., Ltd. (Shanghai, China)	Standard substance for content analysis (purity ≥ 98%). CAS No. 116-53-0

**Table 2:** LC-IRMS stability (δ^13^C values and SD in ‰).

Independent replicate run	1	2	3	4	5	6	7	8	9	10	SD
Instrument stability	0.019	−0.000	0.012	0.019	0.020	0.057	0.061	0.048	0.064	0.071	0.026

**Table 3:** Repeatability and within-laboratory reproducibility (δ^13^C values and SD in ‰).

Independent replicate runs	1	2	3	4	5	6	7	8	9	10	SD
Within-laboratory reproducibility	−20.83	−20.81	−20.71	−20.36	−20.83	−20.54	−20.50	−20.00	−20.15	−20.55	0.29
Intra-day repeatability (RYR sample)	−28.20	−28.28	−28.23	−28.26	−27.78	−28.09	/	/	/	/	0.19
Intra-day repeatability (Lovastatin sample)	−9.12	−9.23	−9.19	−9.18	−9.11	−9.22	/	/	/	/	0.05

**Table 4:** Interlaboratory reproducibility using a lovastatin standard and two market-collected RYR products (δ^13^C values and SD in ‰).

Sample Information	Lab 1	Lab 2	Lab 3	SD
Lovastatin working standard (LRS)	−9.20	−9.57	−9.45	0.19
RYR Sample 1	−26.11	−26.47	−26.38	0.19
RYR Sample 2	−30.12	−30.47	−30.26	0.18

**Table 5:** δ^13^C summaries for the reference populations.

Group Name	Sample Size (*n*)	Min δ^13^C (‰)	Max δ^13^C (‰)	Range δ^13^C (‰)	Mean ± SD δ^13^C (‰)	Median δ^13^C (‰)	Unscaled MAD (‰)
RYR	45	−32.73	−26.68	6.05	−29.74 ± 1.13	−29.52	0.74
Lovastatin	23	−10.17	−6.87	3.30	−8.33 ± 0.80	−8.16	0.47

**Table 6:** One-sided upper tolerance limits.

Coverage	Confidence Level	δ^13^C Threshold (‰)	Nominal Exceedance Probability (%)
90%	95%	−27.86	4.76
95%	95%	−27.38	1.82
99%	95%	−26.47	0.19

## Data Availability

The supporting tables and method-validation information are provided in the Supplementary Materials. The datasets generated during and/or analysed during the current study are available from the corresponding author on reasonable request.

## Supplementary Materials

The following supporting information can be downloaded at: https://www.mdpi.com/article/10.3390/foods15203677/s1, Table S1 (sample information), Tables S2 and S3 (pretreatment optimisation), Figure S1 (residual-solvent check), Table S4 (mixture accuracy), Tables S5–S7, Figure S2 and Equations (S1) and (S2) (reference-population statistics and threshold derivation), Table S8 (controlled blends), Table S9 (Pretreatment effects on isotope measurements), Table S10 (analytical range), Table S11 (between-group variation and leave-one-group-out thresholds) and Sections S1–S8.

## Abbreviations

The following abbreviations are used in this manuscript:

Abbreviations	Substance
2-MBA	2-methylbutyric acid
EA-IRMS	elemental analyser–isotope ratio mass spectrometry
LC-IRMS	liquid chromatography–isotope ratio mass spectrometry
MK	monacolin K
LRS	lovastatin reference/working standard
MTBE	methyl tert-butyl ether
ICC	intraclass correlation coefficient
RYR	red yeast rice
UTL	upper tolerance limit
VPDB	Vienna Pee Dee Belemnite
VPDB-LSVEC	carbon isotope scale normalized using VPDB and the LSVEC lithium carbonate reference material
