# Supplementary material for: Fragment-Specific Stable Carbon Isotope Analysis for Screening the Authenticity of Red Yeast Rice Products
Source: Foods. 2026 Oct 16;15(20):3677. doi: 10.3390/foods15203677 (PMC13704346; doi:10.3390/foods15203677)
Supplement: Supplementary file 1 [file foods-15-03677-s001.zip › foods-4578751-supplementary.pdf]

**Supporting Information**

# Fragment-Specific Stable Carbon Isotope Analysis for Screening the Authenticity of Red Yeast Rice Products

Zhuying Wu[a, b], Daobing Wang[b] , Di Feng[b], [c], Yuan Shuang[d], Shanshan Zhao[b], Xueyao Li[b], Yuhan Tang[b], Guohui Li[b], Lanqing Wang[b], Qiding Zhong[b,] *, Cuiying Zhang[a]*

[a] College of Biotechnology, Tianjin University of Science and Technology, Tianjin 300457, China

[b] Technology Innovation Center of Light Industrial Consumer Goods Quality and Safety for State Administration for Market Regulation, Sinolight Technology Innovation Center Co., Ltd., Beijing 100015, China

[c] College of Chemistry and Life Science, Beijing University of Technology, 100 Pingyuan Park, Chaoyang District, Beijing 100022, China

[d] Sinolight Inspection and Certification (Taiyuan) Co., Ltd.,, Shanxi 030400, China

*Corresponding authors:

Qiding Zhong, Sinolight Technology Innovation Center Co., Ltd., Beijing 100015, China.

E-E-mail: fa_center@163.com

Cuiying Zhang, College of Biotechnology, Tianjin University of Science and Technology, Tianjin 300457, China

E-mail: cyzhangcy@tust.edu.cn

## S1. Sample information

Table S1 records sample information and the $\delta^{13}C$ values of hydrolysis-derived 2-MBA. The authentic RYR set comprises R01–R45 from ten manufacturers (Suppliers A–J); pharmaceutical batches are L01–L23, and commercial RYR products retain C01–C20 for Figure 8. Zhejiang powder samples R01–R03 came from Suppliers A, A and B, respectively. C10, C14 and C15 exceeded the investigational −26.47‰ threshold, making them screening positives relative to the investigated Chinese reference population, without establishing lovastatin addition or its proportion. The RYR and pharmaceutical products represented distinct production batches, with no batch sampled twice; manufacturing dates across the combined sample set spanned November 2024 to April 2026. The pharmaceutical lovastatin batches (L01–L23) were manufactured between November 2024 and January 2026. RYR and pharmaceutical products were stored at room temperature in a dry place and protected from light, either in their original sealed packaging or, after subsampling, in sealed polyethylene containers. Storage duration was not recorded. Samples were equilibrated to room temperature immediately before pretreatment. The MK column records label claims unless explicitly identified as a measured value; R26's measured content for the blend experiment was determined according to the China light-industry standard (QB/T 2847-2023, in Chinese) [52]. Lovastatin and 2-MBA working standards were stored in a refrigerator at 4–8 °C.

**Table S1.** Sample information and 2-MBA $\delta^{13}C$ values

| No. | Sample Type | $\delta^{13}C$ of 2-MBA (‰) | MK content in RYR (label claim unless stated) | Dosage form | Raw material/product | origin | Manufacturer / supplier |
|---|---|---|---|---|---|---|---|
| R01 | RYR | -28.11 | 3.00% | powder | raw material | Zhejiang | A |
| R02 | RYR | -30.95 | 3.00% | powder | raw material | Zhejiang | A |
| R03 | RYR | -29.52 | 3.00% | powder | raw material | Zhejiang | B |
| R04 | RYR | -30.57 | / | powder | raw material | Guangdong | C |
| R05 | RYR | -31.18 | / | powder | raw material | Guangdong | C |

| No. | Sample Type | δ13C of 2-MBA (‰) | MK content in RYR (label claim unless stated) | Dosage form | Raw material/product | origin | Manufacturer / supplier |
|---|---|---|---|---|---|---|---|
| R06 | RYR | -30.60 | / | powder | raw material | Guangdong | C |
| R07 | RYR | -30.26 | 0.60% | powder | raw material | Zhejiang | C |
| R08 | RYR | -29.45 | 1.50% | powder | raw material | Zhejiang | C |
| R09 | RYR | -29.26 | 1.50% | powder | raw material | Zhejiang | C |
| R10 | RYR | -30.55 | 1.50% | powder | raw material | Zhejiang | C |
| R11 | RYR | -29.25 | 3.00% | powder | raw material | Zhejiang | B |
| R12 | RYR | -29.15 | 3.00% | powder | raw material | Zhejiang | A |
| R13 | RYR | -29.37 | 3.00% | powder | raw material | Zhejiang | B |
| R14 | RYR | -29.33 | 5.00% | powder | raw material | Zhejiang | B |
| R15 | RYR | -29.21 | 5.00% | powder | raw material | Zhejiang | C |
| R16 | RYR | -29.50 | 5.00% | powder | raw material | Zhejiang | C |
| R17 | RYR | -29.67 | >3% | powder | raw material | Hubei | D |
| R18 | RYR | -29.19 | >3% | powder | raw material | Hubei | D |
| R19 | RYR | -29.85 | >3% | powder | raw material | Hubei | D |
| R20 | RYR | -28.60 | / | powder | raw material | Hubei | D |
| R21 | RYR | -29.20 | / | powder | raw material | Hubei | D |
| R22 | RYR | -28.76 | / | powder | raw material | Hubei | D |
| R23 | RYR | -28.68 | >3% | powder | raw material | Beijing | E |
| R24 | RYR | -29.12 | >3% | powder | raw material | Beijing | E |
| R25 | RYR | -28.23 | / | powder | raw material | Shandong | F |
| R26 | RYR | -30.41 | 5.22% | powder | raw material | Shandong | F |
| R27 | RYR | -30.08 | / | powder | raw material | Shandong | F |
| R28 | RYR | -30.43 | / | powder | raw material | Sichuan | G |
| R29 | RYR | -27.16 | / | powder | raw material | Sichuan | G |
| R30 | RYR | -30.73 | / | powder | raw material | Sichuan | G |
| R31 | RYR | -29.14 | / | powder | raw material | Sichuan | G |
| R32 | RYR | -29.53 | / | powder | raw material | Zhejiang | H |
| R33 | RYR | -32.73 | / | powder | raw material | Zhejiang | H |
| R34 | RYR | -30.29 | / | powder | raw material | Zhejiang | H |
| R35 | RYR | -29.27 | / | capsule | product | Guizhou | I |
| R36 | RYR | -30.40 | / | capsule | product | Shanxi | J |
| R37 | RYR | -30.59 | 0.60% | powder | product | Zhejiang | B |
| R38 | RYR | -29.46 | 0.60% | powder | product | Zhejiang | A |
| R39 | RYR | -31.97 | >3% | powder | product | Zhejiang | B |
| R40 | RYR | -30.05 | 0.66% | decoction piece | product | Zhejiang | B |
| R41 | RYR | -26.68 | 1% | capsule | product | Zhejiang | A |
| R42 | RYR | -31.53 | 0.50% | capsule | product | Zhejiang | A |
| R43 | RYR | -30.18 | / | tablets | product | Zhejiang | A |
| R44 | RYR | -30.82 | 0.4%~0.65% | capsule | product | Zhejiang | A |
| R45 | RYR | -29.18 | 0.25% | capsule | product | Zhejiang | B |

| No. | Sample Type | δ13C of 2-MBA (‰) | MK content in RYR (label claim unless stated) | Dosage form | Raw material/product | origin | Manufacturer / supplier |
|---|---|---|---|---|---|---|---|
| L01 | Lovastatin | -8.08 | / | capsule | pharmaceutical lovastatin | Anhui | / |
| L02 | Lovastatin | -8.04 | / | capsule | pharmaceutical lovastatin | Sichuan | / |
| L03 | Lovastatin | -8.37 | / | capsule | pharmaceutical lovastatin | Sichuan | / |
| L04 | Lovastatin | -8.25 | / | capsule | pharmaceutical lovastatin | Sichuan | / |
| L05 | Lovastatin | -8.64 | / | capsule | pharmaceutical lovastatin | Sichuan | / |
| L06 | Lovastatin | -8.13 | / | capsule | pharmaceutical lovastatin | Henan | / |
| L07 | Lovastatin | -8.09 | / | capsule | pharmaceutical lovastatin | Jiangsu | / |
| L08 | Lovastatin | -7.82 | / | capsule | pharmaceutical lovastatin | Sichuan | / |
| L09 | Lovastatin | -8.70 | / | capsule | pharmaceutical lovastatin | Sichuan | / |
| L10 | Lovastatin | -8.64 | / | tablets | pharmaceutical lovastatin | Shandong | / |
| L11 | Lovastatin | -7.69 | / | capsule | pharmaceutical lovastatin | Anhui | / |
| L12 | Lovastatin | -7.55 | / | capsule | pharmaceutical lovastatin | Anhui | / |
| L13 | Lovastatin | -7.64 | / | capsule | pharmaceutical lovastatin | Anhui | / |
| L14 | Lovastatin | -7.30 | / | capsule | pharmaceutical lovastatin | Jiangsu | / |
| L15 | Lovastatin | -6.87 | / | capsule | pharmaceutical lovastatin | Jiangsu | / |
| L16 | Lovastatin | -8.01 | / | tablets | pharmaceutical lovastatin | Shandong | / |
| L17 | Lovastatin | -10.17 | / | capsule | pharmaceutical lovastatin | Sichuan | / |
| L18 | Lovastatin | -10.04 | / | capsule | pharmaceutical lovastatin | Jiangsu | / |
| L19 | Lovastatin | -9.72 | / | capsule | pharmaceutical lovastatin | Anhui | / |
| L20 | Lovastatin | -8.90 | / | capsule | pharmaceutical lovastatin | Anhui | / |

| No. | Sample Type | $\delta^{13}C$ of 2-MBA (‰) | MK content in RYR (label claim unless stated) | Dosage form | Raw material/product | origin | Manufacturer / supplier |
|---|---|---|---|---|---|---|---|
| L21 | Lovastatin | -8.36 | / | capsule | pharmaceutical lovastatin | Jiangsu | / |
| L22 | Lovastatin | -8.16 | / | tablets | pharmaceutical lovastatin | Shandong | / |
| L23 | Lovastatin | -8.43 | / | capsule | pharmaceutical lovastatin | Sichuan | / |
| C01 | Commercial RYR products | -29.72 | / | capsule | product | Shanxi | Retail |
| C02 | Commercial RYR products | -29.16 | 0.25% | capsule | product | Zhejiang | Retail |
| C03 | Commercial RYR products | -29.40 | 0.01% | capsule | product | Guizhou | Retail |
| C04 | Commercial RYR products | -31.78 | 2% | capsule | product | Beijing | Retail |
| C05 | Commercial RYR products | -31.41 | 3.74% | powder | product | Zhejiang | Retail |
| C06 | Commercial RYR products | -29.11 | 0.91% | powder | product | Zhejiang | Retail |
| C07 | Commercial RYR products | -29.94 | 1.69% | powder | product | Zhejiang | Retail |
| C08 | Commercial RYR products | -29.98 | 0.10% | capsule | product | Shanxi | Retail |
| C09 | Commercial RYR products | -26.72 | / | capsule | product | Sichuan | Retail |
| C10 | Commercial RYR products | -19.89 | 0.007%-0.01% | capsule | product | Guizhou | Retail |
| C11 | Commercial RYR products | -31.42 | 0.19% | capsule | product | Guizhou | Retail |
| C12 | Commercial RYR products | -27.46 | 0.48% | capsule | product | Shandong | Retail |
| C13 | Commercial RYR products | -28.18 | 0.31% | capsule | product | Guangdong | Retail |
| C14 | Commercial RYR products | -18.31 | 0.16% | tablets | product | Hubei | Retail |
| C15 | Commercial RYR products | -15.93 | 0.16% | tablets | product | Hubei | Retail |
| C16 | Commercial RYR products | -31.82 | 2% | capsule | product | Beijing | Retail |
| C17 | Commercial RYR products | -29.97 | 0.50% | capsule | product | Zhejiang | Retail |

| No. | Sample Type | δ13C of 2-MBA (‰) | MK content in RYR (label claim unless stated) | Dosage form | Raw material/product | origin | Manufacturer / supplier |
|---|---|---|---|---|---|---|---|
| C18 | Commercial RYR products | -29.95 | / | powder | product | Sichuan | Retail |
| C19 | Commercial RYR products | -31.91 | 0.25% | powder | product | Zhejiang | Retail |
| C20 | Commercial RYR products | -29.61 | / | powder | product | Sichuan | Retail |

## S2. Evaluation and rationale of sample pretreatment

RYR samples were ground to a powder when necessary. Approximately 5.0 g was placed in a 50 mL centrifuge tube with 30 mL of 6 mol/L hydrochloric acid, ultrasonicated and vortexed to disperse the sample, then hydrolysed in the capped tube at 65 °C for 12 h. After cooling, 10 mL of diethyl ether was added. The mixture was vortexed for 30 s and centrifuged at 5000 r/min for 6 min. The upper organic phase was transferred to another 50 mL tube; extraction was repeated once and the organic phases combined. The extract was mixed with 2 mL of potassium hydroxide in methanol (2 g/100 mL) and vortexed for 2 min. The ether–methanol mixture was adjusted to approximately pH 8 with 2 mol/L aqueous sulfuric acid, using broad-range pH indicator paper to judge the endpoint. This was an operational reading in the extraction mixture. Diethyl ether and methanol were evaporated at 50 °C under a gentle nitrogen stream. The residue was mixed with 3 mL of water and dried at 105 °C, reconstituted with 1 mL of water and dried again at 105 °C, then reconstituted with a further 1 mL of water. After centrifugation at 10,000 r/min for 10 min, the supernatant was collected and mixed with 50 μL of 2 mol/L sulfuric acid to prepare the pre-injection stock solution. A 200 μL aliquot was diluted with 900 μL of water, vortexed, passed through a 0.22 μm hydrophilic membrane filter and stored until analysis. At the LC-IRMS wet oxidation interface, 0.25 mol/L sodium persulfate solution and 0.5 mol/L phosphoric acid solution were each supplied at 150 μL/min.

### S2.1 Basis for the selected hydrolysis conditions

Hydrolysis was intended to cleave the ester attaching the 2-methylbutyryl side chain to monacolin J [24] and recover 2-MBA reproducibly, while limiting isotope effects from processing. Recovery and replicate precision were assessed together. A systematic isotope shift, however, requires a separate material-matched comparison before and after treatment and cannot be established from precision alone.

The acid concentration and sealed-vessel format were based on the amino-acid hydrolysis step using 6 mol/L hydrochloric acid in a sealed tube [33]. That procedure cleaves amide (peptide) bonds at 110 °C for 22 h [33]. Here, the target was the ester linking the 2-methylbutyryl side chain to monacolin J [24]. Its greater susceptibility to acid-catalysed hydrolysis than that of amide bonds [32] provided the rationale for selecting 65 °C for 12 h. These settings were fixed operating conditions, not the result of systematic temperature–time optimisation or of compound-specific kinetic measurements. Recovery and chromatographic observations below support their use in the tested preparations, but do not quantify residual intact ester or establish complete hydrolysis across different sample matrices.

The acidic hydrolysate favours extraction of predominantly non-ionised 2-MBA (aqueous pKa ≈4.8 at 25 °C [34]) into diethyl ether [48]. Acid treatment may also disrupt the starch–protein matrix [49]. These mechanisms explain the operating rationale; their individual contributions were not quantified.

Chemical profiling studies have examined the lactone and hydroxy acid forms of MK in RYR products [9,10]. Their extraction conditions differ from those used here, so their recovery values cannot be transferred directly to this procedure. Both forms

retain the 2-methylbutyryl side chain [9,10], providing a shared structural target for the hydrolysis step.

Potassium hydroxide was added after extraction to form the potassium salt of 2-MBA, reducing volatility during solvent removal and neutralising co-extracted acid [49]. The subsequent adjustment to approximately pH 8 was made before evaporation; an aqueous Henderson–Hasselbalch estimate at pH 8 would place more than 99.9% of 2-MBA in the anionic form (aqueous pKa ≈4.8 [34]). This percentage was not measured in the ether–methanol mixture and cannot be assigned to its operational pH reading; the intended salt formation limits volatilisation during solvent removal. The later sulfuric acid addition prepared the aqueous stock for injection. Sulfuric acid was selected for compatibility with the mobile phase and the persulfate wet oxidation system [36,38,39]. The chosen KOH amount was an operating condition; its sufficiency across matrices and its contribution to injection-solution ionic strength were not quantified separately. Neither a minimum salt load nor complete removal of chloride was established by the extraction procedure.

### S2.2 Extraction solvent volume

The 10 mL solvent volume was a fixed practical setting: approximately 30 mL of hydrolysate in a 50 mL tube left space for mixing with that volume of ether. Solvents and extraction cycles were compared at 10 mL; no comparative volume series was performed.

### S2.3 Selection of extraction solvent

Solvent selection considered immiscibility with water, partitioning of 2-MBA, volatility and chemical stability. Three solvents were compared: n-hexane, methyl tert-butyl ether (MTBE) and diethyl ether. A 15 mg portion of lovastatin was treated by the procedure above, using 10 mL of the candidate solvent per extraction. Each preparation underwent two successive extractions, after which the organic phases were combined for recovery assessment (Table S2). The two extractions were steps within one preparation, not independent experimental replicates. Diethyl ether gave the highest recovery among the tested solvents and was selected for the final workflow. GC was used to measure 2-MBA in the combined organic extracts. Recovery (%) = 100 × measured 2-MBA amount / theoretical 2-MBA amount from the lovastatin portion. The denominator uses the one-to-one molar relationship between lovastatin and released 2-MBA; for mass-based amounts, theoretical 2-MBA mass equals lovastatin mass multiplied by the 2-MBA-to-lovastatin molar-mass ratio. The GC procedure was based on GB 28336—2012 [35], whose specified content assay uses area normalization. To calculate absolute recovery, the content result must be converted to the recovered 2-MBA amount using a defined quantitative calibration or mass/volume balance. The percentages in Tables S2 and S3 are recovery estimates under the reported GC procedure and do not independently establish absolute recoveries.

**Table S2.** 2-MBA recovery by extraction solvent

| Solvent | Recovery Rate (%) |
|---|---|
| n-hexane | 42.6 |
| MTBE | 78.5 |
| Diethyl Ether | 97.6 |

### S2.4 Optimisation of extraction cycles

One, two and three extraction cycles were compared after hydrolysis of 15 mg of lovastatin, with 10 mL of diethyl ether used per cycle. Table S3 reports three replicate recovery values for each cycle count. Two successive extractions gave a mean recovery of 97.6% (RSD, 2.5%; n = 3), while a third gave no material improvement. RSD was the sample standard deviation (denominator n − 1) divided by mean recovery and multiplied by 100. Two cycles were selected on that basis; the three reported replicates should be distinguished from the extraction cycles within a preparation. Recovery was calculated from the GC measurement as described in Section S2.3.

**Table S3.** 2-MBA recovery by extraction cycle

| Extraction cycles | Parallel No. | Recovery rate (%) | Mean recovery (%) | RSD (%) |
|---|---|---|---|---|
| | 1# | 78.6 | | |
| 1 | 2# | 74.6 | 75.6 | 3.4 |
| | 3# | 73.7 | | |
| | 4# | 98.5 | | |
| 2 | 5# | 94.9 | 97.6 | 2.5 |
| | 6# | 99.5 | | |
| | 7# | 95.8 | | |
| 3 | 8# | 97.4 | 97.0 | 1.1 |
| | 9# | 97.7 | | |

## S2.5 Removal of residual solvent

Residual diethyl ether was detected after addition of 2 mL of potassium hydroxide in methanol (2 g/100 mL) and evaporation at 50 °C. The residue was therefore treated with 3 mL of water and dried at 105 °C, then reconstituted with 1 mL of water and dried again at 105 °C. Ether was not detected by gas chromatography (GC) after this second dissolution–drying step under the conditions shown in Figure S1. The GC temperature programme was 40 °C for 3 min, followed by an increase to 100 °C at 5 °C $min^{-1}$ and a 3 min hold, then an increase to 240 °C at 15 °C $min^{-1}$ and a 5 min hold.

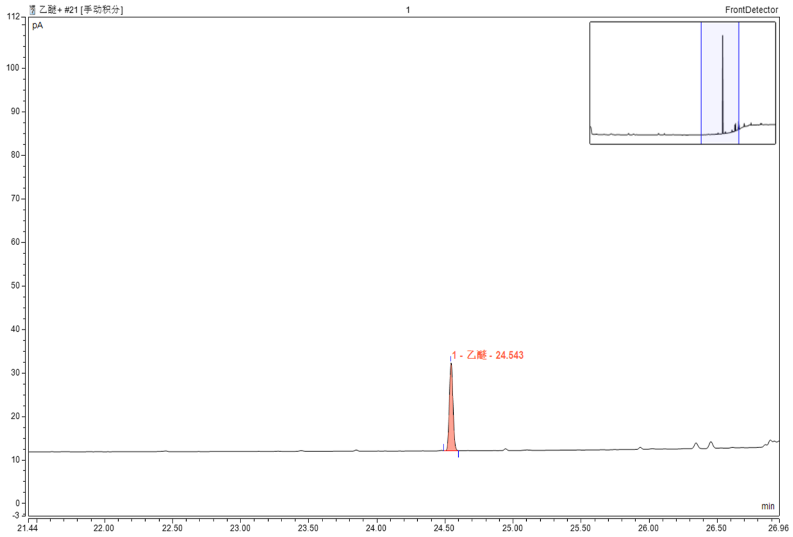


**Figure S1a.** Diethyl ether reference chromatogram

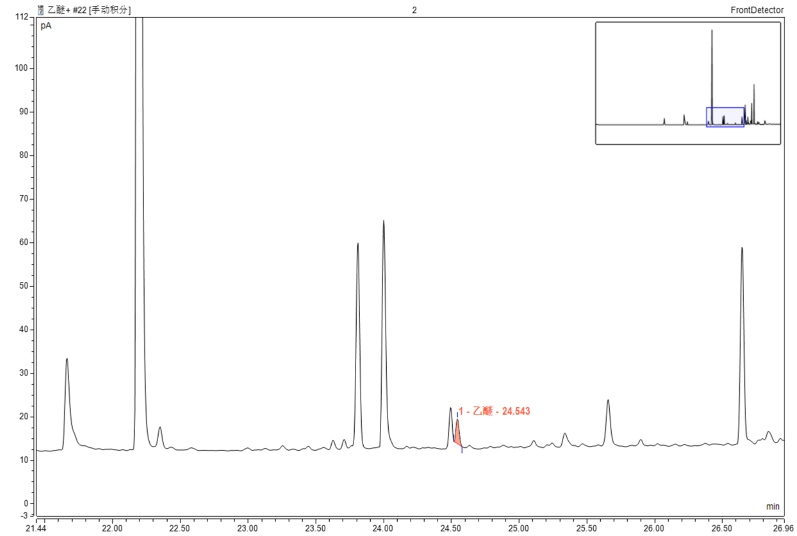


**Figure S1b.** Residual ether after first drying

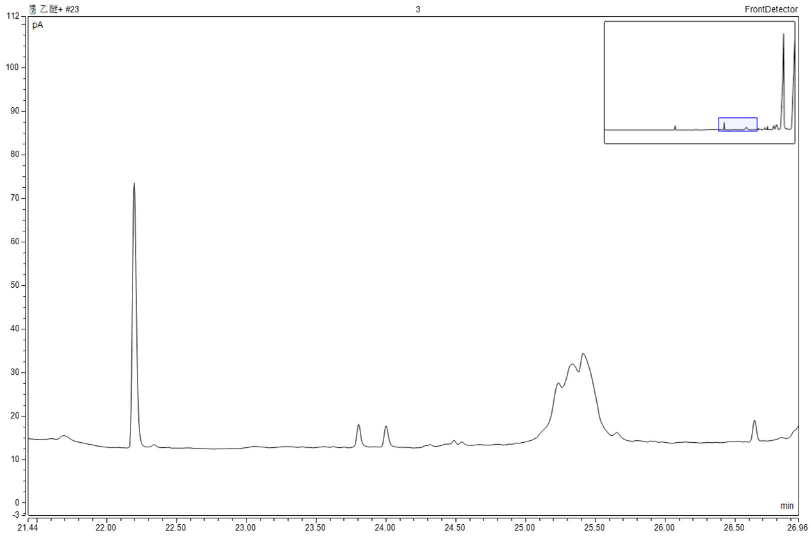


**Figure S1c.** Residual ether after second drying

## S3. Agreement with mass-balance predictions

No compound-specific isotope-certified 2-MBA reference material was used. Agreement with assigned isotope compositions was assessed using mixtures of lovastatin solution (0.0989 mol $L^{-1}$) and 2-MBA solution (0.0990 mol $L^{-1}$) at defined molar ratios, processed by the same pretreatment and LC-IRMS workflow. Each mixture was analysed in duplicate. Measured values agreed with mass-balance predictions ($R^2 = 0.9985$; Table S4). The table reports absolute differences; all were below 0.5‰. The experiment checked agreement with the prepared-mixture predictions; it did not assess accuracy against an independent compound-specific certified value.

Table S4 predictions retain the reported working-standard inputs of −8.45‰ for the lovastatin component and −24.54‰ for 2-MBA on the stated VPDB-LSVEC scale. The measured column contains results from the present analytical sequence: −8.58‰ for 2-MBA released from the lovastatin standard and −24.79‰ for the 2-MBA standard. Assigned inputs and measured endpoints serve different roles and are not substituted for one another. Their differences indicate agreement with the specified model, not independently certified accuracy; assignment uncertainty and calibration transfer were not propagated into the prediction uncertainty.

IAEA-CH-6 anchored calibration at $\delta^{13}C = -10.45$‰ on the VPDB-LSVEC scale (standard uncertainty, 0.03‰; $k = 1$) [40]. The 2-MBA working standards were assigned directly by LC-IRMS measurement relative to IAEA-CH-6. The two-component uncertainty estimate [51] used the ten values in main-text Table 3, which gave a sample SD of 0.28797‰.

Reproducibility and reference-value uncertainty were combined in quadrature, assuming independence and unit sensitivity coefficients, to give a combined standard uncertainty of 0.28953‰. Using a coverage factor k = 2 gave an expanded uncertainty of 0.57906‰, reported as ±0.58‰. The full reproducibility component was retained for triplicate sample means. Working-standard assignment, calibration transfer, scale nonlinearity, pretreatment bias and matrix contributions were not quantified separately; the reported result is limited to this two-component estimate.

**Table S4. Agreement between measured** isotope **values and** mass-**balance** pre**dictio**ns

| Molar ratio (2-MBA:lovastatin) | Predicted Value of $\delta^{13}C$ (‰) | Measured Value of $\delta^{13}C$ (‰) | Absolute difference (‰) |
|---|---|---|---|
| 0:100 | -8.45 | -8.58 | 0.13 |
| 25:75 | -12.47 | -12.25 | 0.22 |
| 50:50 | -16.50 | -16.31 | 0.19 |
| 75:25 | -20.52 | -20.18 | 0.34 |
| 100:0 | -24.54 | -24.79 | 0.25 |

## S4. Statistical analysis and threshold derivation

Triplicate product measurements were averaged to give one observation per production batch. Reference-population distributions were assessed by Shapiro–Wilk tests and Q–Q plots (Figure S2; Table S5). Tests did not reject normality (RYR: W = 0.969, p = 0.260; pharmaceutical lovastatin: W = 0.927, p = 0.092), although the upper-tail observations illustrate the limits of judging distribution shape from small samples. Normality is not established by these checks, particularly at n = 45 and n = 23, respectively. No Grubbs outliers were detected. The RYR mean and sample SD were −29.74‰ and 1.13‰; pharmaceutical lovastatin gave −8.33‰ and 0.80‰.

The one-sided upper tolerance limit (UTL) was calculated as UTL = $\bar{x}$ + ks, where $\bar{x}$ and s are the reference-population mean and sample standard deviation and k is the normal tolerance factor. For confidence 1 − α and coverage p, k is obtained from the non-central t distribution with n − 1 degrees of freedom and non-centrality $z_p\sqrt{n}$, divided by $\sqrt{n}$; $z_p$ is the standard-normal quantile for p. With n = 45 and 95% confidence, k was 1.669, 2.092 and 2.898 for 90%, 95% and 99% coverage, giving UTLs of −27.86‰, −27.38‰ and −26.47‰. The latter was used in the main text.

$$UTL = \bar{x} + k \cdot s \quad (S1)$$

$$k = \frac{t(1-\alpha;\mathrm{n}-1;z_p\sqrt{n})}{\sqrt{n}} \quad (S2)$$

The calculation uses the sample standard deviation (denominator n − 1), as defined in Equations (S1) and (S2): $\bar{x}$ = −29.7376‰, s = 1.1271‰, $z_{0.99}$ = 2.3263 and k = 2.8979, giving UTL = −26.4713‰ (Table S6).

The sample SD uses n − 1 throughout, since the 45 observations represent a sample from a larger reference population. Using an n-denominator SD with the same tolerance factor instead gives −26.51‰ and reduces the achieved confidence to 94.1%. Table S7 shows the effect of this denominator choice on the upper limit.

A distribution-free UTL covering 99% of the population requires 299 observations to attain 95% one-sided confidence. The present n = 45 cannot meet that requirement, so the selected parametric limit depends on the normal model. Shapiro–Wilk results and Q–Q plots are diagnostic checks on that assumption and do not establish it.

The fitted normal distribution gives nominal exceedance probabilities of 4.76%, 1.82% and 0.19% at the 90%, 95% and 99% coverage limits, respectively. These probabilities are model-derived, rather than measured false-positive rates for future products. Observed exceedances in the reference set were 2/45 (4.4%), 2/45 (4.4%) and 0/45 (0%). A chi-square calculation with n − 1 degrees of freedom gave a 95% confidence interval of 0.933–1.424‰ for the sample SD.

A non-parametric bootstrap with 20,000 resamples of 45 observations gave a 95% percentile interval of −27.41‰ to −25.62‰ for the 99%/95% UTL (median, −26.55‰). Omitting R41 (−26.68‰), R29 (−27.16‰), or both gave −26.79‰, −26.69‰ and −27.05‰, respectively (Table S7). This sensitivity to the limited reference set calls for caution when

interpreting results close to the threshold.

With the lovastatin endmember fixed at the assigned working-standard/model input of −8.45‰, rather than the current LD1 measured endpoint of −8.58‰, the modelled LD1 fraction needed to reach the threshold ranged from 1.1% to 25.8% across the 45 RYR reference samples (median, 14.5%). Because this interval is derived from the observed spread of the authentic $\delta^{13}C$ values rather than from a single pair of means, the point estimates quoted in the main text (approximately 15% for the population means and approximately 18% for the R26–LD1 pair) should be read as specific to those particular endmember combinations.

The interval reported above was obtained as follows. For each authentic RYR sample i with measured $\delta^{13}C$ value x(i) (Table S1, R01–R45), the LD1 mass fraction f required for the mixture to reach the threshold was solved from the mass-balance relation $x(i)\cdot(1 - f) + (-8.45)\cdot f = -26.4713$, giving $f = (-26.4713 - x(i)) / (-8.45 - x(i))$. The 45 solutions were then summarised by their range and median. The lower limit is set by the sample with the highest $\delta^{13}C$ value (−26.68‰, R41) and the upper limit by the sample with the lowest value (−32.73‰, R33); the 14.5% median corresponds to a sample close to the reference mean of −29.74‰. The minimum is 1.1445% before rounding (1.1% to one decimal place). Equating this conditional model fraction with LD1 mass fraction assumes comparable molar side-chain yields.

For the 16 commercial products with numerical MK declarations, Spearman's correlation between declared content and $\delta^{13}C$ was $\rho = -0.46$ ($p = 0.072$). Excluding the three screening positives gave $\rho = -0.19$ ($p = 0.54$). C10's label range was represented by its lower endpoint for the rank calculation. The analysis did not detect a statistically significant association; it does not establish independence between declared MK content and isotope composition.

Controlled blends combined R26 (measured MK content, 5.22%; hydrolysis-derived 2-MBA $\delta^{13}C$, −30.41‰) with LD1 lovastatin–dextrin powder (nominal lovastatin content, 5.22%). Predictions use the assigned lovastatin working-standard/model input of −8.45‰; the LD1 endpoint measured −8.58‰ in the current sequence. The eight RYR mass fractions were 0%, 20%, 40%, 60%, 80%, 90%, 95% and 100% (Table S8). Measured $\delta^{13}C$ followed a linear response ($R^2$ = 0.9997, n = 8 levels); absolute differences for the seven levels with numerical predictions were 0.11–0.21‰. Under the equal-side-chain-yield assumption, the model threshold crossing is approximately 18% LD1, or 0.94% (w/w) exogenous lovastatin. This is a pair-specific conditional estimate, not an experimentally established detection limit.

**Table S5.** Reference-population statistics. Skewness and excess kurtosis use bias-corrected estimators (adjusted Fisher–Pearson G1 and G2); uncorrected estimators give slightly different values.

| Population | n | Mean ± SD (‰) | Median (‰) | Unscaled MAD (‰) | Shapiro–Wilk W | p | Skewness | Excess kurtosis |
|---|---|---|---|---|---|---|---|---|
| Authentic RYR | 45 | −29.74 ± 1.13 | −29.52 | 0.74 | 0.969 | 0.260 | 0.07 | 1.23 |
| Pharmaceutical lovastatin | 23 | −8.33 ± 0.80 | −8.16 | 0.47 | 0.927 | 0.092 | −0.83 | 0.88 |

**Table S6.** UTL derivation (99% coverage; 95% confidence)

| Step | Quantity | Symbol or expression | Value |
|---|---|---|---|
| 1 | Number of authentic RYR reference samples | n | 45 |
| 2 | Mean $\delta^{13}C$ of hydrolysis-derived 2-MBA in the reference population | $\bar{x}$ | −29.7376‰ |
| 3 | Sample standard deviation (denominator n − 1) | s | 1.1271‰ |
| 4 | Standard normal quantile for p = 0.99 | $z_p$ | 2.3263 |
| 5 | Non-centrality parameter | $\delta = z_p\sqrt{n}$ | 15.6056 |
| 6 | Quantile of the non-central t distribution | t(0.95; 44; 15.6056) | 19.4398 |
| 7 | One-sided normal tolerance factor | $k = t/\sqrt{n}$ | 2.8979 |

| Step | Quantity | Symbol or expression | Value |
|---|---|---|---|
| 8 | Upper tolerance limit | $UTL = \bar{x} + k \cdot s$ | −26.4713‰ |
| 9 | Threshold reported in the main text | — | −26.47‰ |

**Table S7.** Threshold sensitivity analysis

| Variant | n | Mean (‰) | s (‰) | k | UTL (‰) |
|---|---|---|---|---|---|
| Sample standard deviation (denominator n − 1) — reported | 45 | −29.7376 | 1.1271 | 2.8979 | −26.4713 |
| Population standard deviation (denominator n) | 45 | −29.7376 | 1.1145 | 2.8979 | −26.5078 |
| Excluding the highest observation (−26.68‰) | 44 | −29.8070 | 1.0380 | 2.9058 | −26.7907 |
| Excluding the observation at −27.16‰ | 44 | −29.7961 | 1.0686 | 2.9058 | −26.6910 |
| Excluding both extreme observations | 43 | −29.8686 | 0.9656 | 2.9141 | −27.0546 |
| Non-parametric bootstrap, 95 % percentile interval (20 000 resamples) | 45 | — | — | — | −27.41 to −25.62 |

**Table S8.** Controlled-blend isotope measurements

| Ratio authentic RYR : lovastatin–dextrin (w/w) | Measured $\delta^{13}C$ (‰) | Predicted $\delta^{13}C$ (‰) | Difference (‰) |
|---|---|---|---|
| 0:100 (LD1) | -8.58 | -8.45 | 0.13 |
| 20:80 | -12.72 | -12.84 | -0.12 |
| 40:60 | -17.04 | -17.23 | -0.19 |
| 60:40 | -21.51 | -21.63 | -0.12 |
| 80:20 | -26.23 | -26.02 | 0.21 |
| 90:10 | -28.32 | -28.21 | 0.11 |
| 95:5 | -29.17 | -29.31 | -0.14 |
| 100:0 (R26) | -30.41 | / | / |

R26-derived 2-MBA measured −30.41‰. Predictions use −8.45‰ as the assigned lovastatin working-standard/model input, whereas the 0:100 LD1 preparation measured −8.58‰ in the current sequence. Predictions assume that the nominal RYR:LD1 mass ratio represents the relative hydrolysable side-chain contribution; equal mass contents alone do not verify this assumption for mixed MK acid/lactone forms. Signed differences are predicted minus measured $\delta^{13}C$. Absolute differences of 0.11–0.21‰ refer to the seven rows with numerical predictions. Table S4 instead reports absolute differences directly.

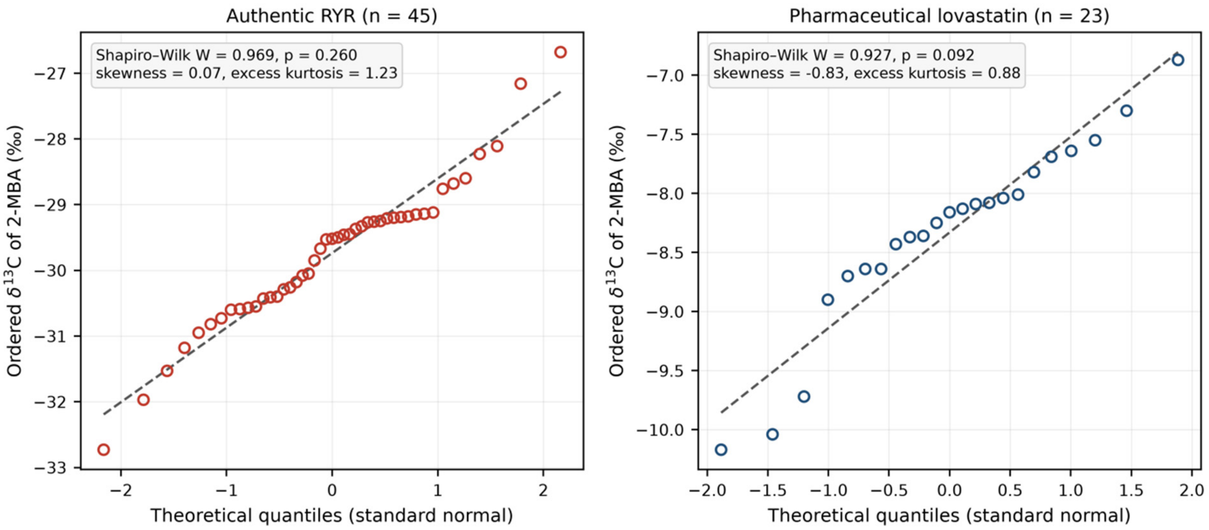


**Figure S2.** Normal Q–Q plots of reference populations

## S5. Scope of the fragment-specific marker

Fragment-specific isotope analysis requires consideration of pretreatment effects. Replicate precision does not estimate a material-matched pretreatment shift (main-text Section 3.2.2). Intact lovastatin standards and their hydrolysis-derived 2-MBA were strongly correlated, but the parent and side chain contain different carbon pools; the correlation cannot independently establish the absence of hydrolysis-induced fractionation. RYR and pharmaceutical samples differed markedly in isotope composition, and controlled blends followed the expected shift between the tested endmembers.

Free 2-MBA and other compounds releasing the same acid on hydrolysis may occur in matrices other than those examined here. Matching retention times for the 2-MBA standard and the lovastatin and RYR hydrolysates support peak assignment (main-text Figure 3), but cannot distinguish these sources of the same compound. Marker specificity therefore depends on the matrix and chromatographic separation. The 23 pharmaceutical batches describe the products sampled; differences in feedstock or manufacturing conditions could alter their isotope range.

## S6. Pretreatment effects on isotope measurements

Two 2-MBA working standards were measured before and after the full pretreatment procedure. Their $\delta^{13}C$ results are summarized in Table S9. Deviations in Table S9 are defined as $\delta^{13}C$ before minus $\delta^{13}C$ after pretreatment.

**Table S9.** $\delta^{13}C$ values of the two 2-MBA working standards before and after sample pretreatment

| Standard | Before pretreatment $\delta^{13}C$ (‰) | After pretreatment $\delta^{13}C$ (‰) | Deviation (‰) |
|---|---|---|---|
| 2-MBA standard 1 | -8.95 | -8.78 | -0.17 |
| 2-MBA standard 2 | -23.59 | -23.65 | 0.06 |

## S7. Working range of the LC-IRMS measurement for 2-MBA

A pigmented RYR material used for food colourant purposes, obtained from the market and not among the commercial products analysed in this study (Table S1), was selected as the spiking matrix in which 2-MBA was not detected under the GC screening conditions. 2-MBA was added at 0.5%, 2.5%, 5.0% and 7.0% (w/w), and taken through the LC-IRMS procedure. Signal intensities ranged from 1209 to 3342 mV, and the measured $\delta^{13}C$ values were −8.55‰, −8.72‰, −8.63‰ and

−8.59‰ (mean −8.62‰; SD across the four concentration levels, 0.07‰; n = 4 levels; denominator n − 1) (Table S10). The four values showed no monotonic change with signal intensity, indicating that no appreciable concentration-related isotope shift was apparent within this tested range. The test used a single matrix and one spiking range; it does not establish the working range at lower analyte contents or in other matrices, and it does not address matrix contributions to the 2-MBA peak. The GC screen does not establish the absence or production origin of marker-releasing compounds.

**Table S10.** Working range of the LC-IRMS measurement: signal intensity and measured $\delta^{13}C$ of 2-MBA added at four levels to a pigmented RYR matrix

| 2-MBA added (%, w/w) | Signal intensity (mV) | Measured $\delta^{13}C$ (‰) | Deviation from the mean (‰) |
|---|---|---|---|
| 0.5 | 1209 | −8.55 | +0.07 |
| 2.5 | 1671 | −8.72 | −0.10 |
| 5.0 | 2516 | −8.63 | −0.01 |
| 7.0 | 3342 | −8.59 | +0.03 |
| Mean (n = 4) | — | −8.62 | — |
| SD (n = 4) | — | 0.07 | — |

## S8. Between-group variation in the reference population and leave-one-group-out thresholds

The 45 authentic RYR observations were grouped by manufacturer (ten groups) and by province (eight groups). One-way analyses of variance did not detect group differences (manufacturer: $F(9,35) = 0.79$, $p = 0.63$; province: $F(7,37) = 0.90$, $p = 0.52$), and the estimated intraclass correlation was approximately zero for both groupings. Leave-one-group-out recalculation of the 99%/95% upper tolerance limit, using the same procedure as Table S6, gave ranges of −26.74 to −26.07‰ across the ten manufacturer omissions and −26.58 to −26.25‰ across the eight province omissions (Table S11). These ranges describe how far the threshold moves when one group at a time is removed from the present reference set; they do not define its uncertainty for other populations, and the grouping factors are unbalanced (Zhejiang supplied 25 of the 45 samples).

**Table S11.** Between-group variation in the authentic RYR reference population and leave-one-group-out upper tolerance limits

| Grouping factor | Groups | n | F (df between, df within) | p | ICC | Leave-one-group-out UTL range (‰) |
|---|---|---|---|---|---|---|
| Manufacturer | 10 | 45 | $F(9,35) = 0.79$ | 0.63 | ≈ 0 | −26.74 to −26.07 |
| Province | 8 | 45 | $F(7,37) = 0.90$ | 0.52 | ≈ 0 | −26.58 to −26.25 |

Reference numbers in this Supporting Information correspond to the reference list in the main manuscript.
